# Cytochromes P460 and *c*′-β: exploiting a novel fold for multiple functions

**DOI:** 10.1007/s00775-025-02102-3

**Published:** 2025-02-26

**Authors:** Hannah R. Adams, Sotaro Fujii, Hans E. Pfalzgraf, Peter Smyth, Colin R. Andrew, Michael A. Hough

**Affiliations:** 1 School of Life Sciences, University of Essex, Wivenhoe Park, Colchester, Essex CO4 3SQ UK; 2 Graduate School of Biosphere Science, Hiroshima University, Kagamiyama 1-4-4, Higashi-Hiroshima, Hiroshima, 739-8528 Japan; 3 Diamond Light Source Ltd., Harwell Science and Innovation Campus, Didcot, OX11 0DE UK; 4 Research Complex at Harwell, Rutherford Appleton Laboratory, Didcot, OX11 0FA UK; 5 Department of Chemistry & Biochemistry, Eastern Oregon University, La Grande, OR 97850 USA

**Keywords:** Heme, Haem, Cross-link, P460, Gas binding, Nitrification

## Abstract

Two related classes of ligand-binding heme *c*-containing proteins with a high degree of structural homology have been identified and characterized over recent decades: cytochromes P460 (cyts P460), defined by an unusual heme-lysine cross-link, and cytochromes *c*′-β (cyts *c*′-β), containing a canonical *c*-heme without the lysine cross-link. The shared protein fold of the cyt P460-cyt *c*′-β superfamily can accommodate a variety of heme environments with entirely different reactivities. On the one hand, cyts P460 with polar distal pockets have been shown to oxidize NH_2_OH to NO and/or N_2_O via proton-coupled electron transfer. On the other hand, cyts *c*′-β with hydrophobic distal pockets have a proposed gas binding function similar to the unrelated, but more extensively characterized, alpha helical cytochromes *c*′. Recent studies have also identified ‘halfway house’ proteins (cyts P460 with non-polar heme pockets and cyts *c*′-β with polar distal heme pockets) with functions yet to be resolved. Here, we review the structural, spectroscopic and enzymatic properties of the cyt P460-cyt *c*′-β superfamily with a view to understanding the structural determinants of their different functional properties.

**Graphical abstract:**

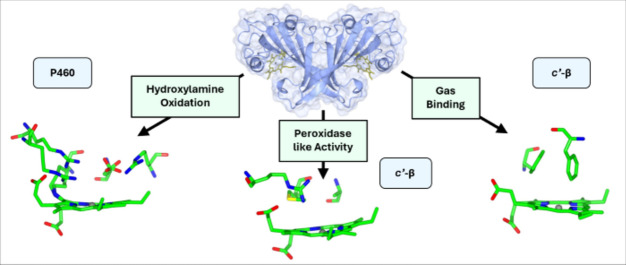

## Introduction

Cytochromes with *c*-heme cofactors occur widely with roles ranging from electron transfer to apoptosis and enzyme reactivity [1, 2]. A relatively recent discovery was a class of evolutionarily related β sheet, ligand binding, mainly dimeric mono-His ligated cytochrome *c* proteins with low sequence and structural homology to other known protein families [3–6]. Based on their spectroscopic properties, this protein family was classified into two groups comprising cytochromes P460 (cyts P460 or *CytL*), named for the characteristic ~ 460 nm absorbance of the Fe^II^ state caused by an unusual heme-Lys cross-link, and cytochromes *c*′-β (cyts *c*′-β or *CytS*) which lack the Lys cross-link, leading to absorption spectra typical of other canonical hemes.

**Fig. 1:**
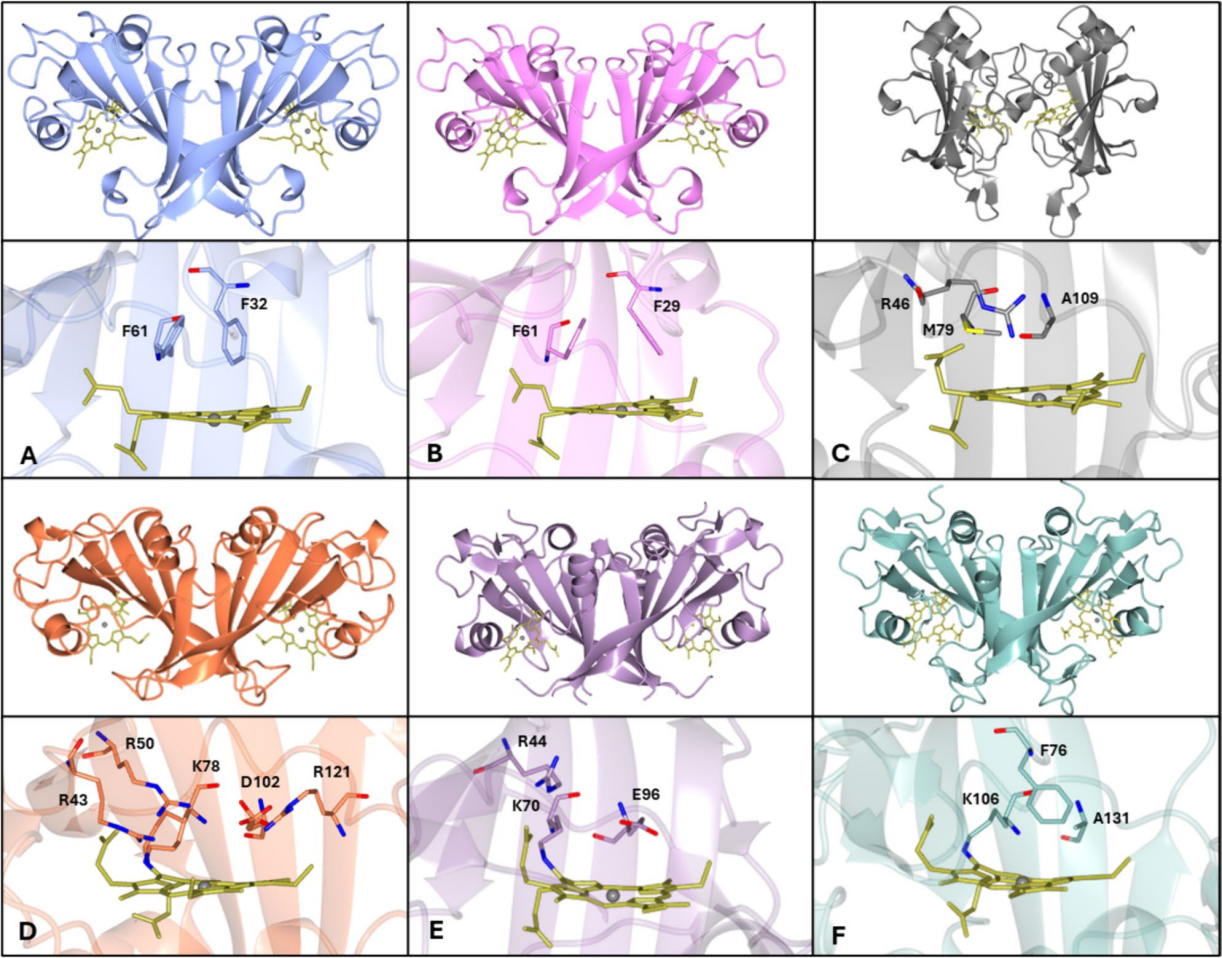
Overall fold (top) and distal heme pocket (bottom) structures of *Mc*CP-β (**A**), *Tt*CP-β (**B**), *Ne*CP-β (**C**), *Mc*P460 (**D**), *Ne*P460 (**E**), and *Ns*P460 (**F**). The overall protein fold and dimeric structure is well maintained within these different proteins, while the active site and surrounding region is highly variable

Remarkably, the very similar overall protein fold of cyts P460 and *c*′-β (but notably distinct from any other protein families) can accommodate different heme active site pockets with entirely different functions (See Graphical Abstract). On the one hand, cyts P460 that contain a carboxylate proton acceptor in addition to their heme-Lys cross-link (Fig. 1D and E) have been shown to catalyze hydroxylamine (NH_2_OH) oxidation to nitric oxide (NO) and/or nitrous oxide (N_2_O). The ability to oxidize NH_2_OH to NO is shared by structurally unrelated multi-heme hydroxylamine oxidoreductases (HAO) that have a Tyr cross-linked P460 heme cofactor, making cyts P460 useful models for the chemistry occurring in these more complex systems [7–9]. On the other hand, cyts *c*′-β with hydrophobic distal pockets (Fig. 1A and B) have a proposed role in binding NO to mitigate oxidative stress, akin to the structurally unrelated α-helical gas-binding cytochromes (cyts *c*′-α) [10–14].

Until recently, these two distinct functional types were considered synonymous with cyt P460 vs cyt *c*′-β dichotomy. However, recent studies have characterized a cyt P460 with a hydrophobic pocket that is unable to oxidize NH_2_OH (Fig. 1F), as well as a cyt *c*′-β with a polar distal sidechain that has peroxidase-like activity (Fig. 1C) [15, 16]. These “half-way house” proteins (of yet to be determined function) reflect the evolution of cyts *c*′-β from cyts P460 supported by genomic data. Understanding the factors that guide this remarkable ability to tune function within a common protein fold has fundamental implications for de novo enzyme design as well as our understanding of protein evolution. Previous excellent reviews have focused on the chemistry of heme P460 containing enzymes [17, 18] and comparing cyt P460 to HAO. Here, we focus on structure–reactivity relationships between cyts P460 and cyts *c′*-β in the context of their functional evolution.

## Occurrence, biological roles, and phylogeny

Cyt P460 was first discovered by Erikson and Hooper in 1972 where it was described as a soluble pigment with a major absorption peak at 463 nm in the reduced-*minus*-oxidized absorption spectrum in extracts from *Nitrosomonas europaea* and thus was designated P460 (referred to as *Ne*P460 in this review) [19]. They noted that it was a heme-containing protein which was able to bind CO. Further purification and characterization was not undertaken until 1990 [20] and a second cyt P460 from the obligate methanotroph *Methylococcus capsulatus* (referred to as *Mc*P460 in this review) was identified by Zahn et al. in 1994 [3]. Cyt *c′*-β was first identified during purification of *Mc*P460 directly from source [3]. Spectroscopic and electrophoretic analysis demonstrated that the separation of three other proteins from *Mc*P460 preceded a UV–visible spectral shift from 460 nm in cell extracts to 450 nm in the purified sample. These were two non-heme containing proteins (61.2 kDa and 26 kDa) and a cyt *c′* (referred to as *Mc*CP-β in this review). The properties of this cyt *c′* were described in 1996 by Zahn et al. [4]. The protein was shown by electron paramagnetic resonance spectra to have a high spin, S = 5/2, heme center and the UV–visible spectra of the ferric and ferrous protein were characteristic of cyts *c*′. However, the redox potential (E_m7_ = -205 mV) was found to be much lower than any previously characterized  cyt *c*′ whose midpoint potentials are positive and range between + 3 mV for *Rhodospirillum rubrum* cyt *c′* and + 202 mV for *Paracoccus denitrificans* cyt *c*′ [14].

The amino acid sequence of *Mc*CP-β showed very low sequence similarity (6–11%) to other known cyts *c′* [6], but when compared to the sequences of cyt P460 from both *M. capsulatus* and *N. europaea,* a higher level of similarity was found (31 and 18% respectively). It was postulated that this high sequence similarity indicated an evolutionary relationship between the cyt *c′* of *M. capsulatus* and cyt P460. Further sequence analysis [21] has supported this hypothesis of an evolutionary relationship and the existence of a new ‘family’ of cytochromes. Furthermore, using secondary structure prediction tools, all the members of this new ‘family’ were predicted to have structures rich in β-sheets in contrast to the typical α-helical structure of all previously characterized cyts *c′,* leading to these new proteins being referred to as cyts *c′*-β (as opposed to cyts *c′*-α).

More recent sequence analysis [22] of cyts *c′*-α, cyts *c′*-β, and cyts P460 clarified the relationship between the three groups of proteins. The cyts *c′*-α were grouped in a separate clade to all the other proteins examined, confirming that they have evolved completely separately to both the cyts *c′*-β and cyts P460. The nesting of the cyts *c′*-β within two separate branches of cyts P460 clearly shows that the cyt *c′*-β evolved from the cyts P460. This is contrary to previous proposals [6] with this discrepancy likely due to the larger number of available sequences and improved phylogenetic methods in the two decades that separate these studies. The observation that none of the organisms within the recent analysis contained both a cyt *c′*-α and a cyt *c′*-β is interesting and suggests that these bacteria never had a cyt *c′*-α in their genome. It could be inferred from this that the evolution of the cyts *c′*-β from the P460s arose due to a need to have a protein which could carry out a role similar to that of the cyts *c′*-α. Despite the clear evolutionary history and the high structural homology between the cyts *c′*-β and cyts P460, there is very little sequence homology between the two groups. Even within each group of proteins, there is low sequence homology: apart from the heme binding CXXCH motif, only 5 residues are completely conserved in cyts P460 and 7 within the cyts *c′*-β. While not fully conserved, the distal heme pocket residues in both the cyts *c′*-β and cyts P460 show reasonable conservation, and where a different residue is present, it is often one of a similar property. This suggests that the type of residue present and its potential ability to interact with ligands is the important factor. As previously shown [21], the cyt P460 and cyt *c′*-β sequences from the latest study are from a wide range of proteobacteria.

**Fig. 2:**
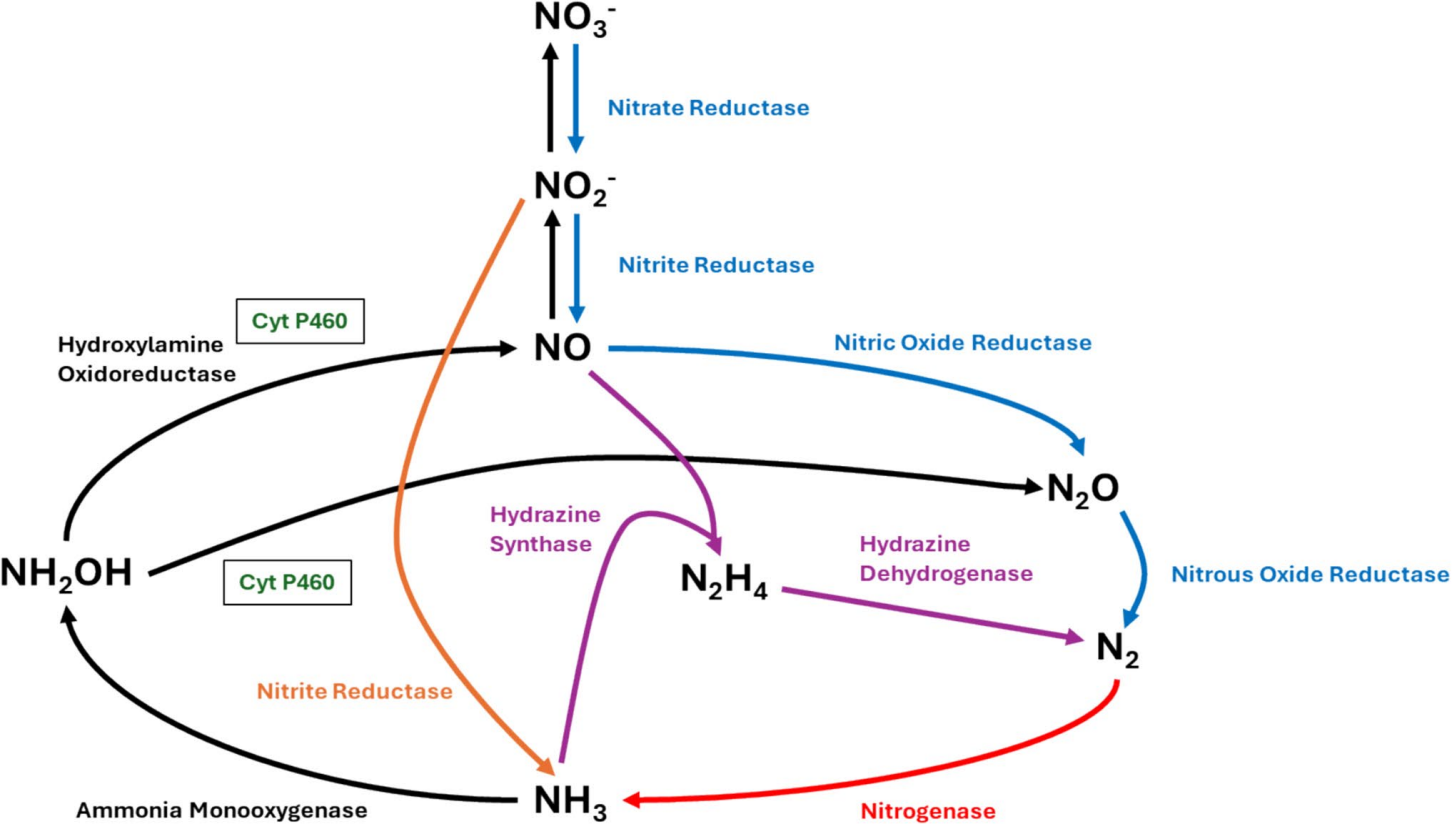
Overview of the reactions within the Nitrogen Cycle highlighting the role of Cyt P460. Blue lines represent denitrification, red lines represent nitrogen fixation, orange lines represent dissimilatory nitrate reduction to ammonium, black lines represent nitrification, and purple lines represent anammox

Microbial metabolism, as a response to modern agricultural methods and the fixed-N saturation of the environment, is the largest source of atmospheric nitrous oxide (N_2_O), a potent greenhouse gas, and ozone depleter [23]. Ammonia-oxidizing bacteria (AOB) are proposed to emit N_2_O either as a byproduct of the nitrification pathway, or as the product of the nitrifier denitrification pathway (Fig. 2). The ammonia oxidation pathway is well studied within AOB but less so in ammonia-oxidizing nonlithotrophic bacteria (ANB), a group of organisms known to aerobically oxidize ammonia to nitrite, but that do not use this as their source of energy [7, 24–28]. The first step in the ammonia oxidation pathway involves the conversion of ammonia to NH_2_OH by either ammonia monooxygenase (AMO) in ammonia oxidizing bacteria or methane monooxygenase (MMO) in methane-oxidizing bacteria. NH_2_OH is then converted to nitric oxide by HAO, allowing NO to be released with the potential to be subsequently oxidized to nitrite abiotically under aerobic conditions. Under anaerobic conditions, N_2_O can be produced directly from NH_2_OH oxidation by cyt P460. Nitrite reductase (NiR) reduces nitrite to nitric oxide which can be reduced to nitrous oxide by nitric oxide reductase (NOR) or cyt P460.

NH_2_OH (generated from oxidation of ammonia) is oxidized within two biological functions (1) energy conservation in the nitrification pathway, and (2) to protect against the inherent toxicity of NH_2_OH. Ammonia-oxidizing bacteria use HAO to capture reducing equivalents from NH_2_OH which are transferred to their electron transfer pathway. NH_2_OH has been shown to transiently accumulate in AOB planktonic or mixed cultures, which potentially could lead to interactions with other key communities involved in the nitrogen cycle [29–31]. The short- and long-term impact of this NH_2_OH accumulation has been tested in AOB without reaching definitive conclusions [32–39].

Nitrite oxidizing bacteria are not able to transform NH_2_OH, but the inhibition of nitrite oxidizers by NH_2_OH might be of relevance when shaping nitrogen cycle communities [29, 40]. In contrast to AOB, methanotrophs cannot capture energy from NH_2_OH oxidation, since they lack the appropriate electron transfer machinery. Consequently, HAO and cyt P460 in methanotrophs may be used to remove buildup of toxic levels of NH_2_OH [41].

**Fig. 3:**
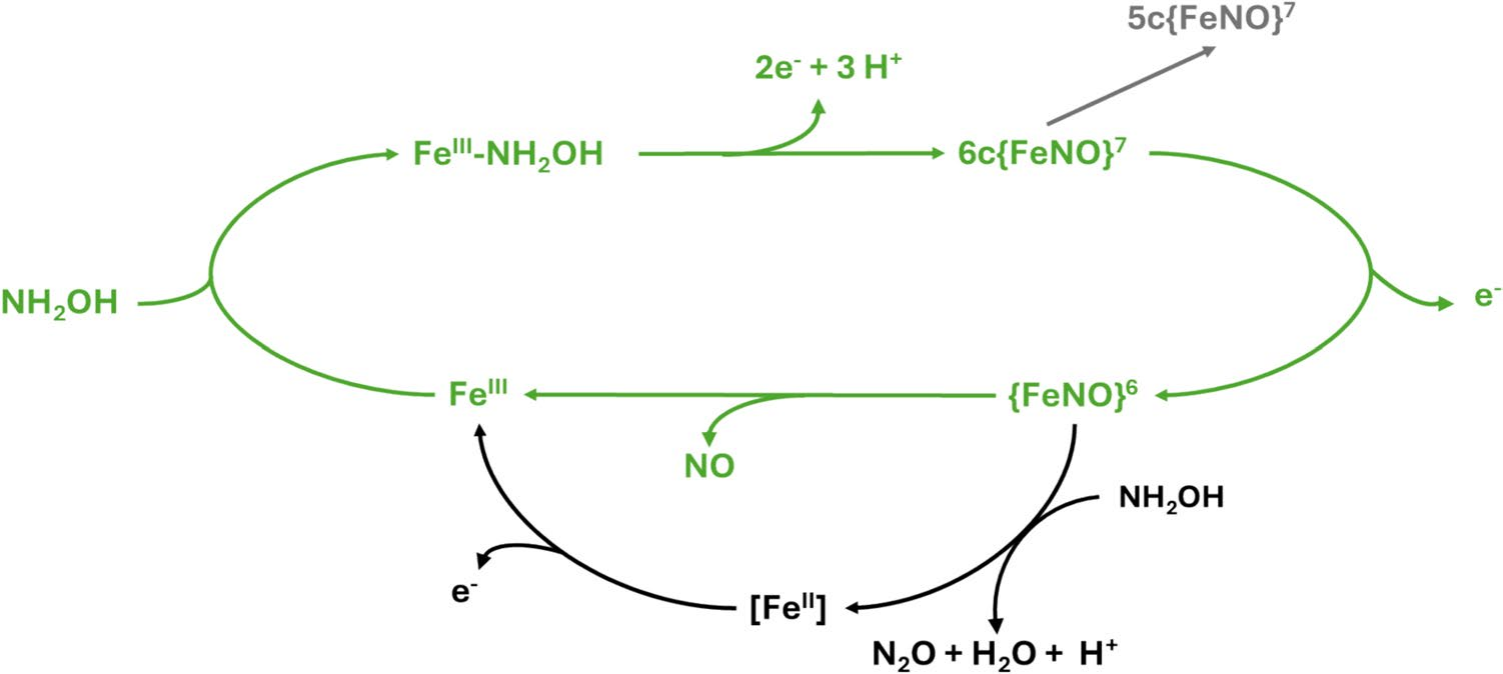
Proposed mechanism of oxidation of hydroxylamine by cyt P460 showing key compounds in the catalytic cycle using Enemark–Feltham notation. Steps in black are those which have been observed only in cyt P460, while those in green have been observed in both HAO and P460. Gray represents an off-pathway 5-coordinate (5c) {FeNO}^7^ species. NH_2_OH binds to the heme of the ferric protein and is oxidized to form an {FeNO}^6^ species via a 6-coordinate (6c) {FeNO}^7^ intermediate. This {FeNO}^6^ species then undergoes nucleophilic attack by a second NH_2_OH to produce N_2_O and H_2_O. The heme is then free to start the cycle over again [18, 42, 43, 45]

The oxidation of NH_2_OH by cyt P460 was originally suggested to produce nitric oxide and nitrite under aerobic conditions [3]; however, recent work has shown that although nitrite is formed under aerobic conditions, the concentration is not stoichiometric to the concentration of NH_2_OH [42]. Under anaerobic conditions, however, it was demonstrated that the enzyme uses four oxidizing equivalents to convert two equivalents of NH_2_OH to N_2_O. This suggests that oxidation of NH_2_OH by cyt P460 contributes to NO and N_2_O emissions from nitrifying bacteria. The proposed mechanism by which this occurs in *Ne*P460 is summarized in Fig. 3, with the Enemark–Feltham notation, {FeNO}^n^, denoting the total number of electrons (n) supplied by the Fe(*d*) and NO(π*) orbitals. NH_2_OH binds to the heme of the ferric protein and is oxidized to form an {FeNO}^6^ product via a 6-coordinate (6c) {FeNO}^7^ intermediate. This {FeNO}^6^ product then undergoes nucleophilic attack by a second NH_2_OH to produce N_2_O and water. The heme is then free to start the cycle over again [15, 42, 43]. The mechanism of NH_2_OH oxidation in the P460 subunit of HAO differs slightly from that of cyt P460 (Fig. 3). While in cyt P460, the NO product remains bound for a sufficiently long period of time to allow the production of N_2_O via interaction with a second molecule of NH_2_OH, whereas in the P460 subunit of HAO, the NO quickly dissociates leading to the production of nitrite by other enzymes instead [17, 44].

The precise biochemical or physiological roles of any cyt *c′* (α or β) have yet to be determined despite their widespread occurrence in nature. However, roles such as cellular defense against nitrosoative stress and in nitric oxide trafficking have been proposed for both cyts *c′-*α [10–12, 14] and cyts *c′*-β such as *Mc*CP-β [46]. Yoshimura and coworkers suggested that the role of cyts *c′-*α could be to sequester and buffer free NO from the periplasm to limit cellular damage due to the protein being able to bind NO that had been generated through denitrification [10, 11]. This was backed up by work from Watmough and colleagues who showed that binding of NO to cyt *c′-*α in vivo was reversible in *P. denitrificans* under physiological conditions [47]. Further work by Cross and coworkers on the cyt *c′-*α from *R. capsulatus* showed that inactivation or knocking out the cycP gene coding for cyt *c′-*α decreased the bacteria’s ability to remove exogenous NO and increased sensitivity to NO [12, 13]. This led to the suggestion that cyt *c′-*α had NO reductase activity; however, work by Choi and colleagues on the cyt *c′-*α from *R. sphaeroides* showed no NO reductase activity leading to the proposal that cyt *c′-*α may play a role in shuttling NO to the membrane where it could be reduced to N_2_O by NOR [48]. In addition, recent characterization of *Ne*CP-β has revealed a distal Arg residue not present in *Mc*CP-β, along with peroxidase-like activity. The presence of an arginine residue above the heme at the active site is reminiscent of the structure of the enzyme chlorite dismutase [49].

## The structures of cyts P460 and cyts *c*′-β

The first indication of the secondary structure for the cyt P460 class of proteins came from sequence analysis and circular dichroism data which indicated a predominately β-sheet fold [21], in contrast to the alpha helical fold that is characteristic of previously studied cyts *c*′ [14]. The first crystal structure to be determined for any protein within the cyt P460-cyt *c'*-β superfamily was that of cyt P460 from *Nitrosomonas europaea* (*Ne*P460), revealing a novel β-sheet fold (Fig. 1E) where each subunit of the dimer contained a heme group with an additional cross-link from a lysine residue [50]. Subsequent crystal structures of native and mutant forms of cyt P460 from *Nitrosomonas* sp AL212 (*Ns*P460) and native *Mc*P460 revealed similar β -sheet folds (Fig. 1F and D), albeit with notable differences in some loops and heme pocket residues (vide infra) (Table 1). The crystal structures of *Mc*CP-β (and subsequently *Thermus thermophilus* cyt *c*′ (*Tt*CP-β)) also confirmed the presence of the β-sheet fold in cyts *c′*-β (Fig. 1A and B). Both the cyts P460 and *c′*-β have a subunit fold comprising a five stranded, antiparallel, twisted type II β-sheet fold, together with several smaller alpha helical features (Fig. 1). This structure is unique to the two families of proteins, as first assessed via a DALI search by Pearson et al. and confirmed at the time of writing this review [50]. The majority of the published structures exist as homodimers, with subtle differences between monomers likely due to the crystalline environment. Early studies on *Ne*P460 suggested the protein existed as either a dimer or trimer [19, 20] with a dimer present in the crystalline state [50]. Whether the other members of the family exist as dimers in solution has yet to be confirmed. Subunit interactions are mostly along the β-sheets, with the hemes of each monomer exposed to solvent on the opposite sides of the dimer interface. This is, however, different for *Ne*CP-β which is thought to potentially exist as a monomer, and if it did form dimers, to have an interface on the opposite side with the hemes facing in toward the middle of the dimer (Fig. 1C). Further work, however, is required to determine conclusively the quaternary structure of *Ne*CP-β. Despite the general similarity of the fold in the majority of the structures, there are, however, a few small differences between them. The C-terminal α-helix found in *Ne*P460, *Ns*P460, and *Ne*CP-β is not found in *Mc*P460, *Mc*CP-β, or *Tt*CP-β [22, 50]. All three published cyt P460 structures have a loop between β-sheets 3 and 4 that reaches the heme in the second monomer which is notably shorter and so does not reach the heme in both *Mc*CP-β and *Tt*CP-β structures. Uniquely *Ne*CP-β has two loops in this part of the structure, one which is similar to the other published cyt *c*′-β structures and a second which loops down away from the rest of the structure, a feature unique to *Ne*CP-β (Fig. 1C). All the cyts *c*′-β do, however, have a loop between β-sheets 1 and 2 which reaches up toward the proximal side of the heme which is considerably shorter in the cyt P460 structures. It is this loop which may prevent *Ne*CP-β from forming a dimer in the same way as the other cyts *c*′-β and cyts P460 [15, 22, 50]. Notably, part of both of these loops are missing in all published *Ne*P460 structures which was attributed to high flexibility in this part of the structure. The relevance of the differences between these loops within the structures has yet to be fully determined.

**Table 1:** Summary of the β-sheet cytochrome structures deposited in the PDB

Cytochrome	Source	Mutation	Ligand	PDB ID
P460	*N. europaea*	–	–	2je3
	*N. europaea*	R44A	–	8gar
	*Nitrosomonas* sp. AL212	–	–	6amg
	*Nitrosomonas* sp. AL212	A131E	–	6eox
	*Nitrosomonas* sp. AL212	A131E	NO	6e17
	*Nitrosomonas* sp. AL212	A131E	NH_2_OH	6eoy
	*Nitrosomonas* sp. AL212	A131Q	–	6eoz
	*Nitrosomonas* sp. AL212	K106L*/*A131E	–	6w6n
	*M. capsulatus* (Bath)	–	–	6hiu
*c′*-β	*M. capsulatus* (Bath)	–	–	6hih
	*M. capsulatus* (Bath)	–	CO	6zsk
	*M. capsulatus* (Bath)	–	NO	7zps
	*M. capsulatus* (Bath)	F32V	–	7zs4
	*M. capsulatus* (Bath)	F32V	CO	7zsx
	*M. capsulatus* (Bath)	F32V	NO	7zsw
	*M. capsulatus* (Bath)	F61V	–	7zrw
	*M. capsulatus* (Bath)	F61V	CO	7zti
	*M. capsulatus* (Bath)	F61V	NO	7zqz
	*T. thermophilus*	–	–	7ead
	*T. thermophilus*	–	–	8brk
	*N. europaea*	–	–	7s5o

## Heme Pockets of cyts P460 and cyts *c*′-β

As is the norm for *c*-type cytochromes, the proximal Fe site of the cyt P460-*c*′-β superfamily is coordinated by the histidine of the CXXCH heme motif [22, 51], while the two cysteines of the motif are covalently bound to the heme. Crystal structures of as-isolated (nominally Fe^III^) forms of cyts P460 and cyts *c*′-β also show a tyrosine residue suitably placed within 3 Å of the histidine to provide a stabilizing hydrogen bond via the main chain carbonyl atom, which may help to maintain the position of the histidine upon ligand binding to the distal site.

Consistent with an enzymatic function requiring proton transfers, the distal pockets of NH_2_OH oxidizing *Ne*P460 and *Mc*P460 contain numerous polar and charged residues (Fig. 1D, E). Because the pockets of *Ne*P460 and *Mc*P460 are exposed to the surface, water molecules can enter the active site and bind to some of these residues. The ability of X-ray crystallography to establish the presence or absence of Fe^III^H_2_O coordination can be complicated by the tendency of ligands present in crystallization solutions to occupy this position and presumably displace water if it was present. For example, a phosphate ion can be seen to be bound to the distal face of the heme in the *Ne*P460 as isolated structure [50]. In contrast to the polar distal pockets of *Ne*P460 and *Mc*P460, the crystal structure of *Ns*P460 reveals a relatively hydrophobic distal pocket (Fig. 1F) and an empty distal Fe site. Although the absence of a water ligand could possibly be due to photoreduction to the Fe^II^ state, this was deemed unlikely since no photoreduction was observed in XAS measurements of Fe^III^ P460 heme cofactors [45]. In the case of cyts *c*′-β, the distal pockets of *Mc*CP-β and *Tt*CP-β contain exclusively hydrophobic residues (Fig. 1A, B), whereas that of *Ne*CP-β (Fig. 1C) contains an Arg side chain. In the case of cyts *c*′-β, all three pockets (Fig. 1A, B, C) appear to be relatively hydrophobic with no evidence of water molecules, although the latter contains an acetate from the crystallization solution. Nevertheless, these surface exposed distal pockets have only the minimum residues necessary to exclude water, while facilitating accessibility to potential heme ligands, such as diatomic gases.

Structure–reactivity investigations of cyts P460 have focused on three distal pocket residues deemed important for NH_2_OH oxidase activity (Lys cross-link, proton accepting carboxylate, and distal capping residue). Table 2 summarizes the identity of these features in structurally characterized cyts P460, together with their equivalents in cyts *c′*-β. The proposed role(s) of these features in cyts P460 and their counterparts in cyts *c*′-β are discussed below [22, 50].

**Table 2:** Heme pocket structural features and NH_2_OH oxidizing ability in native cyts P460 and *c*′-β

	cyts P460			cyts *c′*-β		
	*Ne*P460	*Mc*P460	*Ns*P460	*Ne*CP-β	*Mc*CP-β	*Tt*CP-β
pdbID	2je3	6hiu	6amg	7s5o	6hih	7ead
NH_2_OH oxidation	Yes	Yes	No	No	No	No
Lys cross-link	Yes	Yes	Yes	No	No	No
	Lys70	Lys78	Lys106	Met79	Phe61	Phe61
Distal carboxylate	Yes	Yes	No	No	No	No
	Glu96	Asp102	Ala131	Ala109	Gly82	Gly82
Capping residue	Yes	Yes	Yes	Yes	Yes	Yes
	Phe41^a^	Arg50	Phe76	Arg46	Phe32	Phe29

^a^Presumed capping residue located in a flexible loop not resolved in crystal structure—Identity predicted from amino acid sequence alignments

### Proton accepting carboxylate

A distal pocket carboxylate, positioned to accept and relay protons during NH_2_OH oxidation, is found in catalytically active *Ne*P460 (Glu 96) and *Mc*P460 (Asp 102) (Fig. 1, Table 2). By contrast, this residue is replaced by Ala 130 in the inactive *Ns*P460, with similar substitutions evident in *Ne*CP-β (Ala 109), *Mc*CP-β (Gly 82), and *Tt*CP-β (Gly 82) (Table 2). Mutagenesis studies of *Ne*P460 and *Ns*P460 have concluded that a distal pocket carboxylate must be precisely positioned to accept and relay protons. In the case of *Ne*P460, the fine tolerance for carboxylate positioning is illustrated by the loss of activity in the Glu96Asp mutant. By the same token, only the Ala130Glu mutation (not the Ala130Asp mutation) restored activity in *Ns*P460 [43]. The equivalent Asp 102 residue in *Mc*P460 has its carboxylate located in a similar position to that of Glu 96 due to the harboring β-sheet being closer to the heme than that of *Ne*P460.

### Lys-porphyrin cross-link

The cross-link between the γ-*meso* carbon of the heme and a lysine ε-nitrogen is unique to cyts P460 [52]. This novel posttranslational modification appears to be spontaneous (autocatalytic) upon recombinant expression under aerobic conditions (vide infra) [53]. Intriguingly, anaerobic expression of *Ne*P460 produced an inactive cross-link deficient (CLD) proenzyme that could be restored in vitro by reaction with peroxide [53]. The mechanism of Lys cross-link formation is described in more detail later in this review. All CLD cyt P460 mutants and pro-enzymes characterized to date are inactive, although the presence of the cross-link by itself is not sufficient for catalyzing NH_2_OH oxidation, as evidenced by the inactivity of *Ns*P460 (which has the Lys cross-link but lacks a distal carboxylate containing residue) (Table 2).

The function of the Lys cross-link in cyts P460 has received substantial attention, particularly from Lancaster and co-workers. Crystallographic and enzymatic studies of *Ne*P460 and *Ns*P460 suggest that the Lys cross-link is required to precisely position the distal carboxylate to relay protons during NH_2_OH oxidation (vide supra) [53]. In the case of *Ne*P460, the cross-link also appears to inhibit the formation of an off-pathway 5 co-ordinate (5c) {FeNO}^7^ intermediate in the catalytic cycle (vide infra) which is discussed further later in this review [45].

**Fig. 4:**
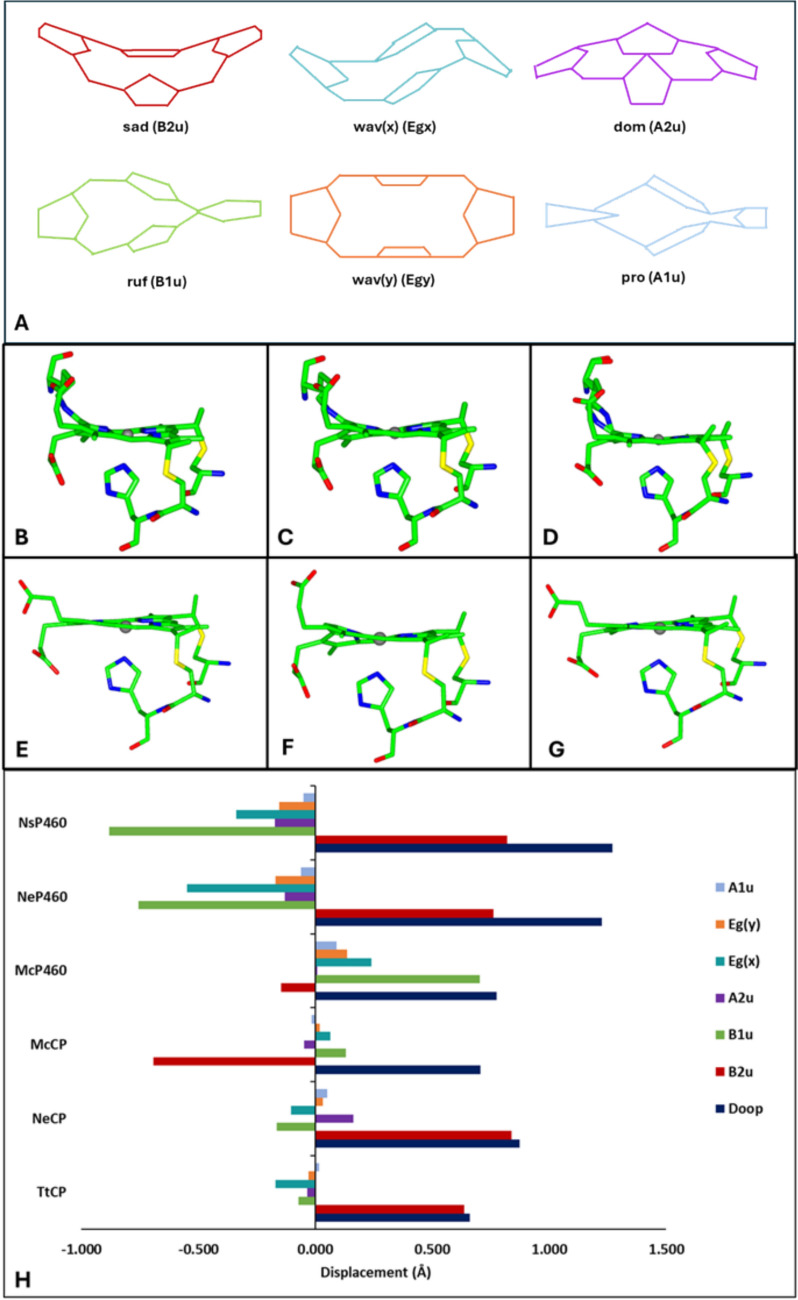
Heme distortions in cyts P460 and cyts *c′*-β. The distortion of the hemes of *Ns*P460 (6amg) (**B**), *Ne*P460 (2je3) (**C**), *Mc*P460 (6hiu) (**D**), *Mc*CP-β (6hih) (**E**), *Ne*CP-β (7s5o) (**F**), and *Tt*CP-β (7ead) (**G**) away from planarity. The different types of heme distortions are shown in Panel A. Graphical representation of the displacement of the heme from planarity for each published wild-type structure (**H**)

The ability to oxidize NH_2_OH to NO (the only known example of heme-bound substrate oxidation in biology) is shared by structurally unrelated multi-heme HAOs that have a Tyr cross-linked P460 heme cofactor. In both cyts P460 and *Ne*HAO, the presence of the P460 cross-link is associated with significant non-planar heme distortions, which may facilitate catalysis, for example by lowering the Fe^III^/Fe^II^ reduction potential. Out-of-plane heme distortions can be quantified using Normal Coordinate Structural Decomposition (NSD) in terms of six distinct displacements from idealized symmetry (Fig. 4) [54]. The most common out-of-plane distortions are doming (*a*2*u*), saddling (*b*2*u*), and ruffling (*b*1*u*) (Fig. 4A). Negative ruffling describes the distortion in which the cross-linked *γ-meso* carbon and the opposite *α-meso* carbon are raised above the plane of the heme. Analysis of the crystal structure of *Ne*HAO using NSD showed that the crosslink to the tyrosine induces severe distortion of the heme away from planarity [44] and this increased level of ruffling distortion was also seen, although to a lesser extent, using the same method with the structures of both *Ne*P460 and *Ns*P460 (Fig. 4F, G) both of which also demonstrated high degrees of saddling [15]. *Mc*P460 (Fig. 4E) was shown [22] to also have a high degree of ruffling but relatively little saddling, while *Mc*CP-β demonstrated high saddling with little ruffling distortions (Fig. 4B). It has been proposed that properties, such as more negative reduction potentials [55] and stronger bonding between the Fe and axial ligand [56], are due to an increased level of ruffling within the heme. For example, the redox potential of *Mc*P460 was − 300 to − 380 mV at pH 7, *Ne*P460 was − 400 mV, while that of *Ns*P460 was -424 mV and the P460 heme of *Ne*HAO was − 260 mV [4, 43, 57]. In contrast, the mid-point potential of *Mc*CP-β, which demonstrated far fewer distortions from planarity, is at − 200 mV at pH 7 (Table 3) [4].

**Table 3:** Reduction potentials of cyts P460 and cyts *c′*-β

	pH	Reduction potential (mV vs NHE)^*a*^	Refs.
*Ne*P460 (wt)	8	− 400 ± 5	[43]
(K70R)	8	− 350 ± 5	[58]
(K70A)	8	− 290 ± 8	[58]
*Ns*P460 (wt)	8	− 424 ± 7	[43]
(A131E)	8	− 428 ± 2	[43]
(K106Y/A131E)	8	− 428 ± 3	[17]
(A131Q)	8	− 406 ± 2	[43]
(A131L)	8	− 381 ± 10	[43]
(A131D)	8	− 388 ± 7	[43]
*Mc*CP-β (wt)	7	− 205	[4]
*Mc*P460 (wt)	7	− 340	[4]

^a^All reported reduction potentials measured using the same methodology of spectroelectrical titrations

In the case of cyts *c*′-β, crystal structures show that the cross-linking lysine is substituted by Phe 61 in *Mc*CP-β and *Tt*CP-β, and by Met 79 in *Ne*CP-β (Fig. 1, Table 2). A more extensive sequence genomic data set for the cyts P460-* c*′-β superfamily suggests that the cross-linking lysine in cyts P460 is substituted by either Phe, Met, Leu, Val, Ile, and Glu with phenylalanine being by far the most commonly occurring [22]. Accordingly, there appears to be a strong preference for hydrophobic residues at this position in cyts *c*′-β, although the significance of an apparent cyt *c*′-β with a glutamate has yet to be investigated. This preference for hydrophobic residues could be due to this type of residue assisting in restricting free water accessing and binding to the active site which in turn allows for faster substrate binding. Recent spectroscopic studies also showed that a Lys cross-link can be introduced in *Mc*CP-β (via the Phe61Lys mutation) and in *Nitrosospira sp.* *NpAv* cyt *c*′-β (*Np*CP-β) (via the Leu105Lys mutation) (Table 4) [53], suggesting that the ability to form a heme-Lys cross-link derives from the shared cyt P460- cyt *c*′-β protein fold.

**Table 4:** Influence of the Lys-porphyrin cross-link and heme redox state (Fe^III^ or Fe^II^) on UV–visible absorption maxima (nm) of cyts P460 and cyts *c*′-β

Protein	Lys cross-link^*a*^	Soret (nm)	CT1 (nm)	Refs.
Fe^III^ cyts *c*′-β				
* Mc*CP-β (wt)	No	399–401	638–640	[4, 22]
(F61K)	No	407		[53]
(F61K)	Yes	439		[53]
* Ne*CP-β (wt)	No	~ 401	642	[61]
* Np*CP-β (wt)	No	400	642	[53]
* Np*CP-β (L105K)	Yes	442		[53]
Fe^III^ cyts P460				
* Mc*P460 (wt)	Yes	419		[4, 22]
* Ne*P460 (wt)	Yes	434–440		[20, 42, 61, 62]
(wt, CLD)	No	404		[53]
(E97A)^b^	Yes	441		[43]
(F41A)	Yes	436		[59]
(F41A, CLD)	No	403		[59]
(F41R)	Yes	442		[59]
(F41R, CLD)	No	402		[59]
(F41W, CLD)	No	403		[59]
(K70A, CLD)^c^	No	402	622–630	[45, 61, 62]
(K70R, CLD)^c^	No	392	638	[62]
(K70Y, CLD)^c^	No	404–406	628–632	[17, 45, 62]
* Ns*P460 (wt)	Yes	440		[43]
(A131D)	Yes	440		[43]
(A131E)	Yes	438		[43]
(A131L)	Yes	441		[43]
(A131Q)	Yes	436		[43]
(K106L/A131E, CLD)	No	402	631	[43]
Fe^II^ cyts *c*′-β				
* Mc*CP-β (wt)	No	431–433		[4, 22]
* Ne*CP-β (wt)	No	432		[61]
* Tt*CP-β (wt)	No	433		[60]
Fe^II^ cyts P460				
* Mc*P460 (wt)	Yes	450–460		[4, 22]
* Ne*P460 (wt)	Yes	460–462		[20, 61, 62]
(K70A, CLD)^*c*^	No	432		[61, 62]
(K70R, CLD)^c^	No	434		[62]
(K70Y, CLD)^c^	No	432		[62]

^a^Lys cross-links in cyts P460 involve K78 (*Mc*P460), K70 (*Ne*P460), or K106 (*Ns*P460) residues. Lys-porphyrin cross-links form spontaneously during aerobic expression of wt cyts P460. Cross-link deficient (CLD) cyts P460 were obtained by preventing the peroxide-based post-translational Lys cross-linking reaction or by removing the cross-linking Lys residue via mutations. Cyts *c*′-β can be engineered to contain a Lys-porphyrin cross-link via the introduction of a Lys residue at the analogous cyt P460 cross-linking position, followed by treatment with Li_2_O_2_ if necessary

^b^E96 referred to as E97 by Smith et. al. (2019) [15]

^c^K70 referred to as K96 by Liew et al. (2020) [61]

### Distal capping residue

A common feature of all structurally characterized members of the cyt *c*′-β-P460 superfamily is a distal “capping residue” (either Phe or Arg) above the heme Fe (Fig. 1, Table 2). The Phe cap variety includes *Mc*CP-β (Phe 32) and *Tt*CP-β (Phe 29), as well as *Ns*P460 (Phe 76). Similarly, Phe 41 is believed to occupy the capping position in *Ne*P460, although it could not be modeled in the crystal structure, presumably due to its location within a flexible and disordered loop [50, 59]. In the case of *Mc*CP-β, we have shown that the Phe 32 distal cap facilitates the dissociation of NO and CO from the heme by means of its aromatic quadrupole that weakens Fe^II^ → XO backbonding and boosts ligand off rates (vide infra) [64]. The non-polar nature of the *Mc*CP-β Phe cap presumably also helps exclude water (and charged species) from the distal pocket, a role also recently proposed for Phe 41 in *Ne*P460 [59]. By contrast, *Mc*P460 contains a charged Arg 50 capping residue, with its guanidinium group pointed toward the heme Fe to interact with ligands (Fig. 1D). A similarly positioned Arg 46 capping residue is also found in *Ne*CP-β, which presumably contributes to its reported peroxidase-like activity (Fig. 1C). We note that a corresponding arginine in *Ne*P460 (Arg 44) does not function as a capping residue, since it points away from the Fe to form a salt bridge with a heme propionate to help stabilize and enforce heme ruffling (Fig. 1E).

### Thermal stability

Temperature dependence circular dichroism measurements demonstrated that *Mc*CP-β denatured in a two-step process at 64 and 94 °C, respectively, while *Tt*CP-β denatured at 116 °C [22, 60]. Thermophilic proteins are generally known to have stabilized structures to adapt to the high temperature environments. X-ray structures of *Tt*CP-β and *Mc*CP-β revealed that the increased interactions at the homo-dimeric interface and the increased number of Pro residues on the loop are responsible for the high stability of *Tt*CP-β [60]. Further sampling as to the stability of cyts *c*′-β will provide insight into the evolutionary diversity of cyts *c*′-β from source organisms that inhabit different types of environments. Notably, *Mc*P460 denatures at 58 °C, which is lower than that of *Mc*CP-β. This reflects the hydrophilic environment around heme in *Mc*P460, while the hydrophobic packing around the heme in *Mc*CP-β contributes to its high stability. These results suggest that differences in heme environments can significantly alter the protein stability even with the same β-sheet folding structures.

## Spectroscopic properties of as-isolated (Fe^III^) and reduced (Fe^II^) forms of cyts P460 and ***c***′-β

### UV–visible absorbance

**Fig. 5:**
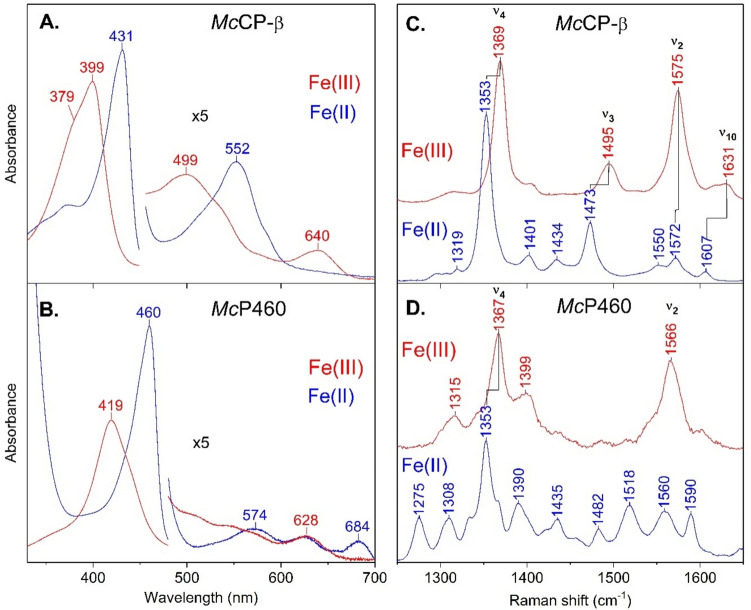
Room-temperature UV–Vis absorbance spectra of *Mc*CP-β (**A**) and *Mc*P460 (**B**) in their Fe^III^ (red trace) and Fe^II^ (blue trace) redox states, together with corresponding resonance Raman spectra (**C** and **D**, respectively) using 407 nm laser excitation (or 442 nm for Fe^II^ *Mc*P460)

Cyts P460 and *c′*-β have distinct absorption features that stem from the presence or absence of the Lys to heme cross-link, as illustrated by the examples of *Mc*P460 and *Mc*CP-β (Fig. 5). Table 4 summarizes λ_max_ values (nm) reported for the most intense heme (Soret) absorbance bands that characterize Fe^III^ and Fe^II^ cyts *c′*-β and P460, as well as the CT1 (Charge Transfer) bands of ferric cyts *c′*-β and cross-link deficient cyts P460 that are markers of Fe^III^ coordination number. Native cyts *c′*-β exhibit absorption features typical of canonical five-coordinate high spin (5cHS) hemes, in line with crystal structures that indicate an empty distal coordination site [4, 22, 53, 60, 61]. The Fe^III^ form of *Mc*CP-β exhibits a Soret absorbance maximum at 399 nm which shifts to 431 nm upon reduction to the Fe^II^ form (Fig. 5, Table 4). Among the weaker absorbance features, the energy of the ferric CT1 band (~ 640 nm) is characteristic of 5cHS Fe^III^ heme, as opposed to 6 co-ordinate (6c) HS Fe^III^ complexes which exhibit CT1 bands closer to ~ 630 nm [4, 22]. Likewise, Fe^III^ *Ne*CP-β displays a broad Soret maximum at 401 nm which shifts to 432 nm in the Fe^II^ state, together with an Fe^III^ CT1 band at 642 nm [61]. Only the Fe^II^ spectrum has been reported for *Tt*CP-β which has a Soret maximum at 433 nm in line with that of the other cyts *c′*-β [60]. Absorbance data for the Fe^III^ form of *Nitrospira* sp. NpAv cyt *c′*-β (*Np*CP-β) (Soret maximum at 400 nm and CT1 band at 642 nm) were also recently reported (Table 4) [53].

Compared to their cyt *c′*-β counterparts, the presence of a Lys-heme cross-link in native cyts P460 results in very different absorbance features, including red-shifted Soret maxima in both the Fe^II^ and Fe^III^ states (Fig. 5, Table 4), in line with trends predicted from time-dependent density functional theory [43]. This shift is believed to be due to a combination of both the lysine cross-link and the distortion of the heme [15, 45]. In the reduced (Fe^II^) state, native cross-linked *Ne*P460 and *Mc*P460 exhibit the eponymous ~ 460 nm heme Soret absorption band (Table 4) [3, 20, 22, 42, 61, 62]. The ferrous P460 cofactor of HAO also exhibits a ~ 460 nm absorbance band, despite possessing a totally different Tyr-heme cross-linking [63]. More variation is observed in the absorption bands of as-isolated (Fe^III^) cyts P460 (Table 4). In particular, the Soret absorption maximum of Fe^III^ *Mc*P460 (419 nm) is notably blue-shifted relative to that of *Ne*P460 (434–440 nm) and *Ns*P460 (440 nm) (Table 4), a difference that we have attributed to *Mc*P460 having a distal H_2_O ligand, although this remains to be confirmed [22]. Assessment of the role of water is challenging, because the distal iron coordination site in the native crystal structure of *Ne*P460 is occupied by a phosphate anion, while the structure of the Arg44Ala variant is coordinated by an acetate molecule, both presumed to be crystallization artifacts [50, 53]. Whereas the presence or absence of H_2_O ligation in canonical high spin Fe^III^ hemes can be inferred from the energy of the CT1 absorbance band, the various weak absorbance bands associated with cyts P460 (see Fig. 5 for example) have yet to be assigned.

Absorbance data have also been reported for a variety of engineered distal pocket mutants of cyts P460 and cyts *c′*-β (Table 4). Significantly, a heme-Lys cross-link was introduced into *Np*CP-β via a Leu105Lys mutation, causing the Fe^III^ Soret maximum of native protein (400 nm) to shift to 442 nm (resembling that of *Ne*P460 and *Ns*P460) (Table 4) [53]. The Fe^III^ absorption maximum of the *Mc*CP-β Phe61Lys mutant after treatment with Li_2_O_2_ (442 nm) is also consistent with Lys-heme cross-link formation (Table 4), further demonstrating that heme-Lys cross-link formation is intrinsic to the shared cyt P460-cyt *c′*-β- fold [59]. Distal pocket mutants of *Ne*P460 and *Ns*P460 that retain the Lys-porphyrin cross-link exhibit Soret maxima similar to those of the wt proteins (Table 4) [43, 59]. On the other hand, cross-link deficient (CLD) versions of *Ne*P460 and *Ns*P460 (obtained by mutating the cross-linking Lys, or by preventing the peroxide-dependent post translational Lys cross-link formation) exhibit absorbance spectra more in keeping with canonical *c*-hemes [17, 43, 45, 53, 59, 62]. With the exception of the *Ne*P460 Lys70Arg variant, many of these CLD cyts P460 show absorbance features typical of 6cHS Fe^III^ heme (~ 630 nm CT1 band) as opposed to 5cHS Fe^III^ coordination (~ 640 nm CT1 band) (Table 4). RR spectra provide additional evidence of 6cHS Fe^III^ populations in CLD cyts P460 (vide infra).

### Resonance Raman (RR) spectra

Cyts P460 and cyts *c′*-β exhibit distinct RR spectra (Fig. 5, Table 5), influenced in part by the presence or absence of the Lys to heme cross-link, as well as the electronic properties of the heme Fe and its environment. Currently available RR data for cyts *c′*-β are for wt and distal pocket mutants of *Mc*CP-β [22], which like other canonical heme proteins, feature several relatively intense “porphyrin marker bands” at vibrational frequencies characteristic of the Fe oxidation state, coordination number, and/or spin state. Room-temperature samples of as-isolated (ferric) *Mc*CP-β exhibit porphyrin marker RR bands, ν_4_ (1369 cm^−1^), ν_3_ (1495 cm^−1^), ν_10_ (1631 cm^−1^) (Fig. 5, Table 5), typical of 5cHS Fe^III^ heme, and consistent with room-temperature UV–Vis and low-temperature crystallographic data that indicate an empty distal coordination site [22]. Similar RR features are observed for the distal pocket *Mc*CP-β mutants, Phe32Val, and Phe61Val (Table 5). Reduced (ferrous) *Mc*CP-β exhibits porphyrin RR bands characteristic of a 5cHS Fe^II^ heme, ν_4_ (1353 cm^−1^), ν_3_ (1473 cm^−1^), and ν_10_ (1607 cm^−1^), along with the axial ν(Fe–His) stretching mode (219 cm^−1^), a frequency somewhat lower than that of alpha helical cyts *c′*-α (~ 230 cm^−1^), suggesting that *Mc*CP-β has a weaker proximal Fe–His bond. RR spectra were also recently reported for Fe^II^CO and Fe^II^NO complexes of *Mc*CP-β [64] to examine the influence of distal pocket aromatic quadrupoles on heme-ligand binding (vide infra).

**Table 5:** Porphyrin marker RR vibrations (ν_4_, ν_3_, ν_10_)^*a*^ for Fe^III^ and Fe^II^ forms of cyts *c*′-β and cyts P460

Protein	Lys cross-link	Soret λ_max_ (nm)	RR λ_ex_ (nm)	ν_4_ (cm^−1^)	ν_3_ (cm^−1^)	ν_10_ (cm^−1^)	Refs.
Fe^III^ cyts *c*′-β							
* Mc*CP-β (wt)	No	399	407	1369	1495	1631	[22]
(F32V)	No		407	1369	1495	1634	[22]
(F61V)	No		407	1369	1495	1634	[22]
Fe^III^ cyts P460							
* Mc*P460 (wt)	Yes	419	407	1367			[22]
* Ne*P460 (wt)	Yes	440	405	1359			[53]
(wt)	Yes	440	405	1364			[53]
(wt, CLD)^*b*^	No	404	405	1369	1488	1616	[53]
(F41A)	Yes	436	405	1367			[59]
(F41A, CLD)^*b*^	No	403	405	1367	1485	1614	[59]
(F41R, CLD)^*b*^	No	402	405	1368	1488	1612	[59]
(F41W, CLD)^*b*^	No	403	405	1367	1495	1611, 1633	[59]
(R44A, CLD)^*b*^	No	403	405	1367	1487	1618	[53]
(K70Y, CLD)^c^	No	406	405	1372	1501		[45]
Fe^II^ cyts *c*′-β							
* Mc*CP-β (wt)	No	431	442	1353	1473	1607	[22]
Fe^II^ cyts P460							
* Mc*P460 (wt)	Yes	460	442	1352			[22]
* Ne*P460 (K70Y, CLD)^*c*^	No	432	405	1356	1473		[45, 62]

^a^proposed vibrational assignments from RR measurements of room-temperature solutions using laser excitation wavelengths, λ_ex_ (nm) near heme Soret absorption maxima, λ_max_, (nm)

^b^cross-link deficient (CLD) protein obtained by preventing the post-translational Lys cross-linking reaction

^c^CLD protein obtained by removing the cross-linking Lys residue via mutation

RR data for cyts P460 have been reported for native forms of *Mc*P460 (Fe^III^ and Fe^II^ states) and *Ne*P460 (Fe^III^ state), as well as for CLD versions of *Ne*P460 obtained by mutating the cross-linking Lys or by inhibiting the post-translational cross-link formation in wt or variant proteins (Table 5). [22, 43, 45, 53]. The presence of a P460 cross-link lowers the overall porphyrin symmetry relative to cyts *c′*-β, leading to an increased number of porphyrin vibrations of similar intensities, such that the corresponding porphyrin marker bands of P460 cofactors are less well defined (Fig. 5). Nevertheless, the most intense RR bands of *Mc*P460 in the Fe^III^ state (1367 cm^−1^) (observed with 407 nm excitation) and the Fe^II^ state (1352 cm^−1^) (observed with 442 nm excitation) are reminiscent of ν_4_ oxidation state marker bands [22] (Table 5). Another relatively intense RR band exhibited by the Fe^III^ state of *Mc*P460 (1566 cm^−1^) was tentatively assigned as the ν_2_ spin-state marker band (Fig. 5) [22]. Lancaster and coworkers reported porphyrin RR modes for Fe^III^ *Ne*P460 (obtained with 405 nm excitation) [43, 53] that differ somewhat from those of Fe^III^ *Mc*P460 obtained under similar conditions (407 nm excitation) [22]. In particular, the range of ν_4_ frequencies reported for Fe^III^ *Ne*P460 (1359 – 1364 cm^−1^) are somewhat lower than that of Fe^III^ *Mc*P460 (1367 cm^−1^) (Table 5), suggesting that some photoreduction of *Ne*P460 may have occurred. Putative ν_3_ (1504 cm^−1^) and ν_10_ (1615 cm^−1^) modes—not observed for Fe^III^ *Mc*P460—were also reported for Fe^III^ *Ne*P460 (the latter attributed to out-of-plane heme distortions) [53]. However, the correspondence of these RR assignments to canonical hemes is unclear, since the proposed ν_3_ 1504 cm^−1^ frequency of Fe^III^ *Ne*P460 is typical of 5cHS heme, whereas the proposed ν_10_ 1615 cm^−1^ frequency is typical of a different (6cHS) coordination. RR measurements of Fe^III^ *Ne*P460 using 459 nm excitation [45] appear to show a different intensity pattern relative to data obtained at 405 nm, although the frequencies of RR bands were not reported. We also note that RR spectra of the Fe^II^ P460 cofactor of *Ne*HAO differ from those of ferrous *Mc*P460 [63]. The precise reasons for these differences are unclear, but could point to differences in P460 porphyrin conformation, symmetry, and/or axial ligation. Future studies of cyt P460 heme complexes should help to solidify the structural interpretation of RR bands.

Because CLD forms of *Ne*P460 contain a canonical *c*-heme, their Fe redox state, spin-state, and coordination number can be readily inferred from their porphyrin marker band RR frequencies. In their ferric state, all the CLD *Ne*P460 proteins exhibit ν_4_ bands indicative of Fe^III^ heme (1367–1372 cm^−1^), similar to that of *Mc*CP-β (1372 cm^−1^) (Table 5) [45, 53, 59, 64]. However, in contrast to *Mc*CP-β proteins, which exhibit ν_3_ (1495 cm^−1^) and ν_10_ (1631–1634 cm^−1^) marker bands typical of 5cHS Fe^III^ (empty distal site), most of the RR spectra reported for CLD versions of *Ne*P460 (from wt, Arg44Ala, Phe41Ala, and Phe41Arg) exhibit lower frequency ν_3_ (1485–1488 cm^−1^) and ν_10_ (1611–1618 cm^−1^) modes that are typical of 6cHS coordination, suggesting the presence of a distal H_2_O ligand. In the case of Phe41Tyr *Ne*P460, the CLD version exhibits a split ν_10_ mode (1611 and 1633 cm^−1^), indicative of a 6cHS/5cHS mixture. Absorbance data for the ferric Lys70Tyr variant (628 nm CT1 band) suggest a 6cHS site (Table 4), whereas the ν_3_ RR frequency (1501 cm^−1^) suggests 5cHS (Table 5). Overall, it appears that in contrast to native cyts *c′*-β, the distal pockets of many CLD cyts P460 are sufficiently polar to allow H_2_O ligation. Meanwhile, the extent to which spectroscopic markers (including UV–Vis absorbance and RR) can establish the presence or absence of Fe^III^H_2_O coordination in native cyts P460 is an ongoing question.

### Electron paramagnetic resonance (EPR)

Cryogenic solutions of as-isolated (Fe^III^) cyts P460 and cyts *c′*-β exhibit EPR parameters consistent with high spin (HS) Fe^III^ heme (Table 6). Early EPR spectra of as-isolated *Ne*P460 displayed a spectrum representative of a HS species with g-values of 5.91, 5.63, and 1.99. Low-temperature EPR analysis of *Mc*P460 showed features representative of a single HS species with g1 = 6.18, g2 = 5.57 and g3 = 1.99 similar to that of the as isolated *Ne*P460 [20] and the ‘P460 fragment’ from *Ne*HAO (Table 6) [63]. The originally described EPR spectrum of *Mc*P460 [3] possibly represents a degraded form of the enzyme.

**Table 6:** EPR parameters for cyts P460 and cyts *c′*-β in the absence of ligands

Protein	pH	Temp (K)	Species	g┴g1	g2		g||g3	Refs.
Cyt P460								
* Mc*P460 (wt)	7	10		6.17	5.57		1.99	[22]
* Ne*P460 (wt)	7	4		5.91	5.63		1.99	[20]
(wt, CLD)	8	12	Major	6.02	5.54		1.99	[59]
Matured Protein	8	12		6.50	5.06		1.97	[59]
(K70Y, CLD)	8	12		5.78	–		1.98	[45]
* Ns*P460 (wt)	8	10	Major	6.39	5.13		1.97	[43]
			Minor	6.00	5.52		1.99	
(A131E)	8	10	Major	6.40	5.14		1.97	[43]
			Minor	6.00	5.51		1.99	
(A131Q)	8	10	Major	6.51	5.12		1.97	[43]
			Minor	6.03	5.53		1.99	
(A131L)	8	10	Major	6.40	5.11		1.98	[43]
			Minor	6.00	5.48		1.99	
(A131D)	8	10	Major	6.40	5.12		1.97	[43]
			Minor	6.03	5.50		1.99	
(K106L/A131E, CLD)	8	10	Major	6.04	5.65		1.99	[17]
			Minor	6.40	5.50		2.00	
Cyt *c′*-β								
*Mc*CP-β (wt)	8	10	Major	6.29	5.46		1.98	[22]
			Minor	6.00	5.34		1.98	
(F32V)	8	10	Major	6.34	5.46		1.98	[64]
			Minor	5.90	5.32		1.98	
(F61V)	8	10	Major	6.30	5.46		1.98	[64]
			Minor	5.89	5.34		1.98	
*Ne*CP-β (wt)	7	12	Major	6.13	5.53		1.99	[61]
			Minor	6.53	–		–	
*Np*CP*-*β								
(L105K)	8	12		6.63	5.07		1.96	[53]

The ferric EPR spectra of *Mc*CP-β suggest the existance of two pure HS species (S = 5/2), the proportions of which do not change with varying pH [22]. Previous work by Zahn et al. also suggested two HS species were present; however, they also suggested that at pH 4, there was a small population of intermediate spin (IS) state (S = 3/2) at around 15%. Cyts *c′*-α have been shown to exist in the ferric state as either pure HS or a quantum mechanical admixture (QS) mixture of HS/IS with the proportion of HS increasing at more alkaline pH, which fits with the proposal of a IS population in the pH 4 samples [4]. This however is not consistent with more recent data [22] where multiple data sets at different concentrations all suggest HS species. EPR spectra of Phe32Val and Phe61Val *Mc*CP-β variants showed a mixture of two HS Fe^III^ species similar to that of the wt protein but with different rhombicities. The only other available EPR spectrum of a cyt *c′*-β, *Ne*CP-β at pH 7, also indicated the presence of two HS species with reported g values of 6.13, 5.53, and 1.99 for the major species and 6.53 for the minor species [61]. More in-depth analysis of the Fe^III^ heme centers of these proteins using EPR-based methods has yet to be carried out.

Interestingly, the EPR spectra for *Ns*P460 also demonstrate two components like the cyts *c′*-β with g values of 6.39, 5.13, and 1.97 for the major species and 6.00, 5.52, and 1.99 for the minor species (Table 6) [15]. It was originally suggested that the two species were due to two conformations of a phenylalanine residue in the distal pocket, but this was not experimentally verified. It has now been suggested that they may be due to two different heme conformations, one being more ruffled and the other more planar [59]. As was seen in the UV–visible spectra, mutating the cross-linking residue in both the *Ne*P460 Lys70Tyr and the *Ns*P460 Lys106Leu/Ala131Glu CLD mutants changed the EPR spectrum. The *Ne*P460 Lys70Tyr mutant exhibited an *S* = 5/2 signal with *g* values of 5.78 and 1.98 in the resting Fe^III^ state which were suggestive of an increased heme symmetry compared to the wt *Ne*P460 [45]. The *Ns*P460 CLD mutants all display two components like the wt protein; however, the signals are less rhombic.

## Ligand-binding reactions of cyts P460

**Fig. 6:**
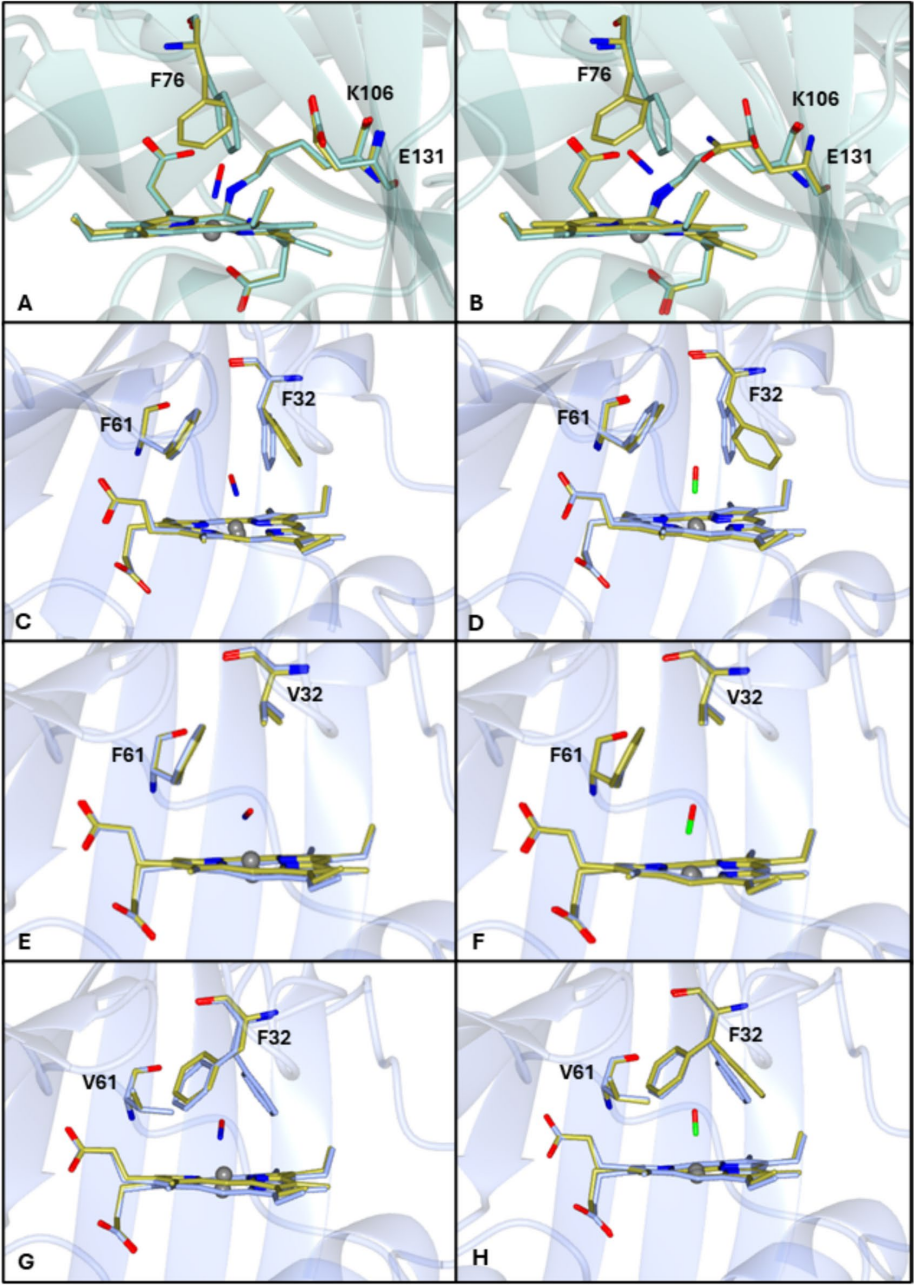
Ligand bound structures (gold) of cyts P460 and *c′*-β in comparison to their ligand free states (green – *Ns*P460/blue—*Mc*CP). NO (6e17) (**A**) and NH_2_OH (6eoy) (**B**) bound Ala131Glu *Ns*P460 demonstrates movement of Phe 76 to allow ligands to bind to the distal face of the heme. NO bound *Mc*CP (7zps) (**C**), Phe32Val *Mc*CP (7zsw) (**E**), and Phe61Val *Mc*CP (7zqz) (**G**) and CO bound *Mc*CP (6zsk) (**D**), Phe32Val *Mc*CP (7zsx) (**F**), and Phe61Val *Mc*CP (7zti) (H) also demonstrate the interaction of the capping Phe residues with movement of Phe 32 upon ligand binding in wt *Mc*CP (**C**, **D**).and the Phe61Val mutant (**G**, **H**). Phe/Val 61 does not show any movement or ligand interaction in either the wt or mutant *Mc*CP

Heme–ligand complexes of cyts P460 have been investigated by spectroscopy and X-ray crystallography to (1) identify characteristic UV–visible absorption features (for use in monitoring kinetics and ligand affinities), (2) investigate the impact of heme pocket mutations on reactivity, and (3) compare the reactivity within the cyt P460-cyt *c′*-β superfamily. Initial spectroscopic studies were carried out on CO, N_3_^−^, CN^−^, and NH_2_OH complexes of *Ne*P460 and/or *Mc*P460. Subsequent spectroscopic data were reported for heme complexes of *Mc*P460, *Ne*P460, and *Ns*P460 (Tables 7, 8), focusing on intermediates within proposed catalytic mechanism for NH_2_OH oxidation (Fe^III^NH_2_OH, {FeNO}^6^, {FeNO}^7^). Dissociation constants (and some kinetic parameters) are available for some Fe^III^NH_2_OH and {FeNO}^6^ species (Table 9). Crystal structures have also been reported for the {FeNO}^7^ and Fe^III^NH_2_OH complexes of the Ala131Glu variant of *Ns*P460 (Fig. 6) [43].

**Table 7:** UV–visible absorption maxima for ligand complexes of cyts *c*′-β and P460

Complex		Lys crosslink	λ_max_ (nm)				Refs.
Cyts *c′*-β							
Fe^III^CN							
*Ne*CP-β	wt	No	419	536			[61]
Fe^II^CO							
*Mc*CP-β	wt	No	420	536	561		[4]
			418	533	560		[64]
	F32V	No	417				[64]
	F61V	No	417				[64]
*Ne*CP-β	wt	No	419	535	564		[61]
*Tt*CP-β	wt	No	419				[60]
Fe^II^O_2_							
*Mc*CP-β	wt^*a*^	No	414	539	572		[64]
5c{FeNO}^7^							
*Mc*CP-β	wt	No	396	533	562		[64]
	F32V	No	394	533	562		[64]
	F61V	No	396	533	562		[64]
*Tt*CP-β	wt	No	395				[60]
6c{FeNO}^7^							
*Mc*CP-β	wt	No	417	542	574		[22]
	F32V	No	414	541	574		[22]
	F61V	No	416	541	573		[22]
Cyts P460							
Fe^III^CN							
*Mc*P460	wt	Yes	435				[3]
*Ne*P460	wt	Yes	457				[20]
		Yes	442				[62]
Fe^III^N_3_							
*Mc*P460	wt	Yes	432				[3]
Fe^III^NH_2_OH							
*Ne*P460	wt	Yes	443			635	[20]
			445	561		633	[42]
	F41A	Yes	446	570		639	[59]
	K70Y	No	410	525	563		[17]
*Ns*P460	K106L/A131E	No	406	526	563		[65]
{FeNO}^6^							
* Ne*P460	wt	Yes	455	554	603	652	[42]
		Yes	455		596	650	[17]
	K70Y	No	419	532	565		[17]
* Ns*P460	K106L/A131E	No	417	529	563		[65]
Fe^II^CO							
* Ne*P460	wt	Yes	446				[19]
			448				[20]
			448		620	688	[62]
			448				[61]
*Mc*P460	wt	Yes	435				[3]
5c{FeNO}^7^							
*Ne*P460	wt	Yes	455	535	584	642	[45]
6c{FeNO}^7^							
*Ne*P460	wt	Yes	452	550	608	665	[45]
	K70Y^b^	No	415	540	580		[45]
		No	415	536	576		[17]
* Ns*P460	K106L/A131E^b^	No	415	538	573		[65]

^a^Undergoes rapid autoxidation to the ferric state

^b^Evidence for subsequent formation of a minor 5c{FeNO}^7^ component from the appearance of 395 nm shoulder (Vilbert 2018)

**Table 8:** EPR parameters for Fe^III^ and Fe^II^ ligand complexes of cyts *c*′-β and P460

Complex	pH	Temp (K)	Species	g⊥g1	g2	g||g3	Refs.
Fe^III^NH_2_OH							
* Ne*P460 (wt)	8	10		2.75	2.28	1.54	[43]
* Ns*P460 (wt)	8	10		2.84	2.25	1.44	[43]
(A131E)	8	10		2.86	2.27	1.46	[43]
(A131Q)	8	10		2.78	2.28	1.49	[43]
(A131L)	8	10		2.80	2.27	1.46	[43]
(A131D)	8	10		2.86	2.25	1.44	[43]
Fe–NO							
* Mc*CP (wt)	4	10	5c{FeNO}^7^	2.02	2.01	1.99	[64]
	7	10	5c/6c{FeNO}^7^	2.02	2.01	1.99	[64]
	10	10	6c{FeNO}^7^	2.01	2.00	1.98	[64]
(F32V)	4	10	5c{FeNO}^7^	2.02	2.01	1.98	[64]
	7	10	5c/6c{FeNO}^7^	2.02	2.01	1.98	[64]
	10	10	6c{FeNO}^7^	2.01	2.00	1.98	[64]
(F61V)	4	10	5c{FeNO}^7^	2.02	2.00	1.98	[64]
	7	10	5c/6c{FeNO}^7^	2.02	2.00	1.99	[64]
	10	10	6c{FeNO}^7^	2.01	1.99	1.99	[64]
*Ne*P460 (wt)	8	10	5c{FeNO}^7^	2.10	2.03	2.01	[45]
	8	10	6c{FeNO}^7^	2.10	2.01	1.98	[45]
(K70Y)	8	8	5c{FeNO}^7^	2.09	2.03	2.01	[45]
	8	8	6c{FeNO}^7^	2.09	2.02	1.98	[45]

**Table 9:** Dissociation and rate constants for Fe^III^ and Fe^II^ ligand complexes of cyts *c*′-β and P460

Complex		Lys crosslink	*K*_d_	*k*_off_	*k*_on_	Refs.
Cyts *c′*-β			(M)	(s^−1^)	(M^−1^ s^−1^)	
Fe^II^CO						
* Mc*CP-β	wt	No	≤ 8 × 10^–9^	0.20	≥ 2.5 × 10^7^	[64]
	F32V	No		0.13		[64]
	F61V	No		0.32		[64]
Fe^II^O_2_						
* Mc*CP-β	wt	No	7.4 × 10^–5^	~ 9000^*a*^	~ 1 × 10^8*a*^	[64]
6c{FeNO}^7^						
* Mc*CP-β	wt	No	≤ 1 × 10^–10^	0.011	≥ 1 × 10^8^	[64]
	F32V	No		0.0045		[64]
	F61V	No		0.016		[64]
Cyts P460						
Fe^III^NH_2_OH			(μM)			
* Ne*P460	wt	Yes	9 ± 1			[42]
		Yes	30 ± 0.7			[59]
	F41A, matured	Yes	22 ± 8			[59]
* Ns*P460	wt	Yes	18 ± 1			[43]
	A131D	Yes	19 ± 7			[43]
	A131Q	Yes	15 ± 3			[43]
	A131L	Yes	12 ± 3			[43]
	A131E	Yes	16 ± 5			[43]
	K106L/A131E	No	7.7 ± 0.3			[65]
{FeNO}^6^			(mM)	(s^−1^)	(M^−1^ s^−1^)	
* Ne*P460	wt	Yes	3.6 ± 0.4	0.35 ± 0.05	9.8 × 10^4*b*^	[59]
	F41A, matured	Yes	16 ± 3	0.30 ± 0.03	1.9 × 10^4*b*^	[59]
	F41R, matured	Yes	86 ± 14			[59]
* Ns*P460	wt	Yes	8 ± 1			[43]
	A131E	Yes	5 ± 1			[17]
	K106L/A131E	No	2.5 ± 0.1			[65]
6c{FeNO}^7^						
* Ne*P460	wt	Yes			> 3.5 × 10^6^	[45]

^a^*k*_on_ and *k*_off_ values estimated from the experimental *K*_d_ value together with reactivity trends in other heme proteins

^b^*k*_on_ value calculated from the ratio of experimental *k*_off_/*K*_d_ values

## Fe^III^NH_2_OH complexes

The formation of an Fe^III^NH_2_OH enzyme:substrate complex is the first step in the proposed catalytic mechanism of cyts P460. In the absence of external oxidant, Fe^III^ *Ne*P460 forms a stable Fe^III^NH_2_OH complex under anaerobic conditions with a ~ 445 nm Soret absorption band (Table 7) and low-spin EPR signal (Table 8) [42]. The formation of a redox stable Fe^III^NH_2_OH complex is rare among heme proteins, and presumably reflects the relatively low reduction potential common to cyts P460 (Table 3). Likewise, stable low-spin Fe^III^NH_2_OH complexes are formed with the P460 centers of *Ns*P460 and a range of distal pocket variants (Table 7). Cross-link deficient mutants of *Ne*P460 (Lys70Tyr) and *Ns*P460 (Lys106Leu/Ala131Glu) also form stable Fe^III^NH_2_OH species with absorption maxima resembling reported values for canonical hemes [66].

Saturation binding curves yielded *K*_d_ values of 9–30 mM for *Ne*P460 (Table 9) [42, 59]. A similar range of *K*_d_ values (8–18 mM) was reported for *Ns*P460, with no significant impact from mutations that removed the Lys cross-link (Lys106Leu/Ala131Glu) or that introduced a distal pocket carboxylate (Ala131Glu and Ala131Asp), amide (Ala131Gln), or branched hydrocarbon (Ala131Leu) at the purported proton relay site (Table 9) [43, 65]. The Ala131Gln *Ns*P460 variant is the only cyt P460 to date for which an X-ray crystal structure of the Fe^III^NH_2_OH complex is available [43]. Each of the four enzyme copies in the structure showed putative NH_2_OH ligands with variable Fe–N–O angles and relatively long Fe–N distances of > 2.7 Å, although the resolution of the data was relatively low (1.97 Å) implying a significant level of uncertainty in these values. Although the Ala131Gln mutation did not introduce catalytic activity to this inactive enzyme, the crystal structure shows that the Gln 131 amide O atom is oriented to accept a hydrogen bond from the N atom of the NH_2_OH ligand. It was proposed that the isostructural Glu 131 present in the active Ala131Glu variant (which has its γ-carboxylate directed away from Fe in the as-isolated structure) could rotate upon NH_2_OH binding to both hydrogen bond and abstract a proton to promote catalysis. On the other hand, the same study reported that *K*_d_ values for the Fe^III^NH_2_OH complexes of Ala131Glu (16 ± 5 mM) and Ala131Gln (15 ± 3 mM) variants are both comparable to that of wt (18 ± 5 mM), suggesting either that hydrogen bonding does not occur in solution, or that it does not significantly impact NH_2_OH-binding affinity. Finally, it has been noted that the Fe^III^NH_2_OH *K*_d_ value of *Ne*P460 (9–30 mM) is relatively high for an enzyme–substrate complex [17]. By comparison, activity assays of HAO have yielded micromolar *K*_m_ values for *Ne*HAO (3.6 µM), *Ks*HAO (4.4 µM), and *m*HAO (1.4 µM) [41, 67, 68] which may indicate that cyt P460 functions to detoxify NH_2_OH only if HAO is saturated.

## Fe^III^NO ({FeNO}^6^) complexes

Another feature of cyts P460 stemming from their low Fe^III/II^ reduction potentials is that they form Fe^III^NO ({FeNO}^6^) complexes that are resistant to reductive nitrosylation. The reaction of NO with Fe^III^ *Ne*P460 generates a Soret absorbance at 455 nm, together with weaker bands at ~ 600 and ~ 650 nm (Table 7). Similar behavior has also been reported for *Ns*P460 variants [59, 65]. Since {FeNO}^6^ complexes are EPR silent, NO binding was ultimately confirmed by FTIR measurements of *Ne*P460 that detected a characteristic N–O-stretching vibration, ν(NO) at 1912 cm^−1^, downshifting to 1871 cm^−1^ with ^15^NO [53]. Significantly, an {FeNO}^6^ complex is an intermediate in the proposed cyt P460 catalytic mechanism (Fig. 3), and has been shown to accumulate (via its ~ 455 nm absorbance) when Fe^III^ *Ne*P460 (and the catalytically competent Ala131Glu variant of *Ns*P460) reacts with NH_2_OH in the presence of external oxidants. Crystallographic characterization of the active Ala131Glu variant provides the only {FeNO}^6^ structure of a cyt P460 to date. The Fe–N–O bond angles (ranging from ~ 110° to ~ 150° in different monomers) suggest that some X-ray induced photoreduction to the {FeNO}^7^ state may have occurred (a phenomenon to which ferric heme proteins are particularly prone). The structure clearly shows the Glu 131 carboxylate pointing away from the Fe, with no evidence of any stabilizing interactions (*e.g.,* hydrogen bonds) between the NO ligand and the distal pocket [43]. That is, hydrogen bonding to the NO ligand does not appear to be required for catalytic activity. Consistent with minimal impact of the engineered Glu 131 residue on NO binding, the *K*_d_ value for the {FeNO}^6^ complex of the Ala131Glu variant (5 ± 1 µM) is quite similar to that of wt *Ns*P460 (8 ± 1 µM) (Table 9). Larger variations in *K*_d_ values were reported between the {FeNO}^6^ complexes of wt *Ne*P460 (5 ± 1 µM) and those of its Phe41Ala (16 ± 3 µM) and Phe41Arg (86 ± 14 µM) variants (Table 9)[59]. The lower NO affinity of the Phe41Arg variant (*K*_d_ value ~ 22-fold higher than wt) was ascribed to the positively charged Arg destabilizing the Fe^II^–NO^+^ ground state in favor of an Fe^III^–NO^•^ excited state (predicted by DFT to have a weaker bond). Although a *k*_off_ value for the Phe41Arg species was not reported, the same study reported *k*_off_ values for the {FeNO}^6^ complexes of wt (0.35 ± 0.05 s^−1^) and Phe41Ala (0.30 ± 0.03 s^−1^) *Ne*P460 (Table 9). These empirical *k*_off_ values enabled predicted *k*_on_ values to be calculated from the *k*_off_/*K*_d_ ratios for wt (9.8 ± 10^4^ M^−1^ s^−1^) and Phe41Ala (1.9 ± 10^4^ M^−1^ s^−1^) (Table 9). It was postulated that the lower *k*_on_ value in the Phe41Ala variant might indicate that H_2_O coordinates to Fe^III^ in the Phe41Ala variant, but not in wt, thereby suggesting a role for the Phe 41 capping residue in excluding solvent from the *Ne*P460 active site. However, this appears at odds with the very similar Fe^III^NH_2_OH *K*_d_ values reported in the same study for Phe41Ala (22 ± 8 mM) and wt (30 ± 0.7 mM) (Table 9).

## Fe^II^NO ({FeNO}^7^) complexes

Spectroscopic studies of *Ne*P460 have shown that 5c{FeNO}^7^ and 6c{FeNO}^7^ complexes can be formed from the reaction of the Fe^II^ state with NO, or from the reaction of HNO with Fe^III^ protein [45]. A 6c{FeNO}^7^ species (λ_max_ 452, 550, 608, 665 nm) is observed initially, followed by conversion to a 5c{FeNO}^7^ end product (λ_max_ 455, 535, 584, 642 nm) (Table 7). Cryogenic EPR measurements of samples frozen at various incubation times confirmed the presence of two distinct *S* = ½ species: a 5c{FeNO}^7^ end product (associated with a characteristic 3-line ^14^N hyperfine pattern) and an initially formed species attributed to the 6c{FeNO}^7^ precursor (with the lack of characteristic 9-line ^14^N superhyperfine splitting ascribed to either a weak or disordered Fe–His bond) [45] (Table 8). Additional X-ray absorption measurements were also consistent with two distinct {FeNO}^7^ species with EXAFS fits yielding Fe–NO distances typical of 5c{FeNO}^7^ (1.74 Å) and 6c{FeNO}^7^ (1.86 Å) heme. Kinetics data revealed that the rate of 6c → 5c {FeNO}^7^ conversion was independent of NO concentration, suggesting that NO remains on the distal face with a His dissociation rate constant, *k*_His-off_ = 5.7 ± 0.2 × 10^–3^ s^−1^ [45]. This rate constant is ~ 100-fold lower than that reported for *Mc*CP-β (*k*_His-off_ = 0.6 ± 0.05 s^-1^) [64], which could indicate that *Ne*P460 has a stronger Fe–His bond than that of *Mc*CP-β.

A 6c{FeNO}^7^ species is an early intermediate in the proposed cyt P460 catalytic cycle, generated via the 2e^−^ oxidation of the Fe^III^NH_2_OH substrate complex (Fig. 3)[45]. Subsequent 1e^−^ oxidation of the 6c{FeNO}^7^ species forms the {FeNO}^6^ intermediate, which then either releases NO or reacts with another NH_2_OH molecule to form N_2_O. Significantly, only the 6c{FeNO}^7^ *Ne*P460 species can be oxidized to the {FeNO}^6^ state, whereas the 5c{FeNO}^7^ counterpart is resistant to oxidation, and therefore constitutes an off-pathway species. Consequently, the relatively slow NO-independent 6c → 5c {FeNO}^7^ rate of *Ne*P460 facilitates catalysis by enabling the rate of oxidation of the on-pathway 6c{FeNO}^7^ intermediate to outcompete formation of the 5c{FeNO}^7^ dead end complex [45].

The Lys70Tyr CLD mutant of *Ne*P460 also exhibited evidence of 6c → 5c {FeNO}^7^ conversion [45]. However, in this case, *two* distinct 6c → 5c {FeNO}^7^ reactions were identified—an NO-independent process, *k*_His-off_ (3.8 ± 0.9 × 10^–4^ s^−1^) (observed when the 6c{FeNO}^7^ complex was generated by manually mixing HNO with the Fe^III^ state), and an NO-dependent bimolecular reaction, *k*_6-5_ (790 ± 80 M^−1^ s^−1^) (observed when the 6c{FeNO}^7^ complex was generated by stopped-flow mixing of excess NO with the Fe^II^ state). In both cases, the initial complex formed within the mixing time and exhibited absorbance features typical of a 6c{FeNO}^7^ canonical heme protein (λ_max_ 415, 540, 580 nm) (Table 7) [45]. Subsequent time-resolved UV–Vis spectra revealed a concomitant decrease in the 415 nm Soret absorbance together with the appearance of a 397 nm shoulder (characteristic of a 5c{FeNO}^7^ population). Although the equilibrium {FeNO}^7^ species in Lys70Tyr was assigned to that of the 5c{FeNO}^7^ complex, its UV–Vis spectrum appears more consistent with a mixture of 5c and 6c populations, suggesting that its Fe–His bond may be stronger than that of wt (for which EPR data indicate essentially complete conversion to an 5c{FeNO}^7^ end product). In line with a stronger Fe–His bond in the Lys70Tyr variant, the NO-independent *k*_His-off_ value of Lys70Tyr (3.8 ± 0.9 × 10^–4^ s^−1^) is an order of magnitude lower than that of wt (*k*_His-off_ = 5.7 ± 0.2 × 10^–3^ s^−1^).

Taken together, the effect of the Lys70Tyr mutation on {FeNO}^7^ coordination points to a possible role for the Lys cross-link in preventing off-pathway 5c{FeNO}^7^ formation. Although the Lys cross-link of *Ne*P460 appears to weaken the Fe–His bond relative to the Lys70Tyr mutant (promoting 5c{FeNO}^7^ formation via the trans effect and speeding up NO-independent release of the His ligand), it also prevents the NO-dependent 6c → 5c {FeNO}^7^ process, thereby limiting the rate at which the 5c{FeNO}^7^ can form. Significantly, *the off-pathway (*5c{FeNO}^7^*) species does not build up in wt NeP460 when NO is present in solution*, allowing the catalytically essential oxidation of the 6c{FeNO}^7^ species to compete. We note that the NO-dependent 6c → 5c {FeNO}^7^ conversion associated with the Lys70Tyr mutation is reminiscent of native cyts *c*′-α, in which a second NO molecule coordinates on the opposite (proximal) heme face to form a proximally bound 5c{FeNO}^7^ end product via a transient dinitrosyl [14]. Thus, it is possible that the Lys cross-link in *Ne*P460 acts to prevent such proximal heme-NO binding. Future studies of cyts P460 will help determine whether the effect of Lys cross-link removal on {FeNO}^7^ reactivity extends beyond the specific Lys70Tyr mutation in *Ne*P460.

## Fe^II^CO complexes

Early UV–Vis studies of *Mc*P460 reported an Fe^II^CO complex with a Soret λ_max_ (435 nm) that is significantly blue shifted relative to the Fe^II^CO complex in *Ne*P460 (448 nm), mirroring the difference in their as-isolated Fe^III^ states: 419 nm (*Mc*P460) vs ~ 440 nm (*Ne*P460) [3]. Although Fe^II^CO complexes are isoelectronic structural analogs of the catalytically relevant {FeNO}^6^ species, no crystal structures of Fe^II^CO cyt P460 species have yet been reported. Interestingly, the absorption maxima for *Ne*P460 are notably different for Fe^II^CO complex (448, 620, 688 nm) and its isoelectronic {FeNO}^6^ counterpart (455, 554, 603, 652) (Table 7), whereas Fe^II^CO and {FeNO}^6^ species of canonical hemes exhibit similar λ_max_ values (~ 418, ~ 535, ~ 565 nm) [17, 19, 20, 42, 61, 62]. The reason for this difference is currently unknown and will hopefully be investigated by future studies.

## Anion complexes

Consistent with its polar distal pocket, early studies of *Mc*P460 reported UV–visible absorption maxima for CN^−^ and N_3_^−^ complexes of both its Fe^III^ and Fe^II^ states (although no spectra were shown) (Table 7)[3, 62]. Anion complexes of other cyts P460 have yet to be reported.

## Ligand-binding reactions of cyts *c*′-β

The influence of the distal heme pocket environment on the coordination chemistry of cyts *c*′-β has been investigated using spectroscopic, kinetic, and crystallographic techniques. Spectroscopic data have been reported for ligand complexes of *Mc*CP-β, *Tt*CP-β, and *Ne*CP-β (Tables 7 and 8), along with crystal structures (Fig. 6) and kinetic data (Table 9) for *Mc*CP-β {FeNO}^7^ and Fe^II^CO complexes. Early characterization of *Mc*CP-β by UV–Vis absorption showed that it was able to bind NO and CO but not ethyl isocyanide, cyanide, or azide [4]. The hydrophobic nature of the *Mc*CP-β distal pocket (containing the Phe cap) presumably accounts for discrimination against anions. Reports of ligand binding to *Tt*CP-β have been limited to CO and NO complexes, although the presence of a hydrophobic distal Phe cap (similar to that of *Mc*CP-β) is also expected to inhibit anion binding [60]. By contrast, the more polar distal pocket of *Ne*CP-β, which contains a distal Arg residue in place of a Phe, is reported to bind cyanide (CN^−^) in addition to CO [61].

Reactions of Fe^II^ *Mc*CP-β with diatomic gas ligands have been extensively studied [4, 64]. Upon reaction with CO, UV–Vis absorption maxima characteristic of an Fe^II^CO complex are observed. Early UV–Vis studies of *Mc*CP-β reported absorption maxima for the {FeNO}^7^ state (λ_max_ 418, 530, 562 nm) that are more typical of an {FeNO}^6^ species [4], suggesting that oxidation of the sample had occurred. Subsequent experiments showed that the {FeNO}^7^ *Mc*CP-β species is stable under strict anaerobic conditions, existing as a pH-dependent equilibrium (p*K*_a_ ~ 7.2) between a 6c{FeNO}^7^ (λ_max_ 415, 540, 565 nm) favored under basic conditions, and a 5c{FeNO}^7^ complex (λ_max_ 395, 540, 560 nm) (His ligand dissociated) at lower pH (Table 7) [64]. Room temperature RR and cryogenic EPR measurements of {FeNO}^7^ *Mc*CP-β confirm the pH-dependent coordination number [64]. Time-resolved UV–Vis measurements of NO binding to Fe^II^ *Mc*CP-β show that the 6c{FeNO}^7^ species forms initially, followed by (NO-independent) His ligand dissociation (*k*_obs_ ~ 0.6 ± 0.05 s^−1^) to form the 5c{FeNO}^7^ population. The unimolecular 6c → 5c {FeNO}^7^ conversion in *Mc*CP-β suggests that its 5c{FeNO}^7^ complex is retained on the distal heme face, in stark contrast to the NO-dependent (bimolecular) process in *Alcaligenes xylosoxidans* cyt *c'*-α (*Ax*CP-α) that is a hallmark of proximal 5c{FeNO}^7^ formation [14]. Although the X-ray crystal structure of the wt *Mc*CP-β {FeNO}^7^ complex (subject to packing constraints) showed only a 6c geometry at pH 6.5, structures of the Phe32Val and Phe61Val variants (which also exhibit pH-dependent 6c{FeNO}^7^-5c{FeNO}^7^ equilibria) revealed elongated Fe-His bonds, consistent with a transition toward distal 5c{FeNO}^7^ species. All three crystal structures were solved at a pH of 6.5 at which, being below the wt *Mc*CP p*K*_a_ of 7.2, it would possibly be expected for them to show a 5c{FeNO}^7^ geometry (as seen in solution studies) not the 6c{FeNO}^7^ geometry that was observed in the wt *Mc*CP-β {FeNO}^7^ complex structure. It is not yet known if other cyts *c´*-β exhibit pH-dependent heme–NO coordination, although a UV–Vis spectrum of the *Tt*CP-β {FeNO}^7^ complex at pH 7.0 reveals a Soret band at 395 nm, together with a 415 shoulder, consistent with a predominantly 5c{FeNO}^7^ population and minor 6c{FeNO}^7^ species [60].

A notable feature of *Mc*CP-β is that gas binding and release are both relatively rapid compared to other heme proteins (Table 9). Stopped-flow measurements of NO and CO binding to Fe^II^ *Mc*CP-β reveal unusually high on rate constants (*k*_on_) that approach the diffusion-controlled limit. Complex formation was mostly complete within the instrument dead time, consistent with *k*_on_ > 1 × 10^8^ M^−1^ s^−1^ for NO and ≥ 2.5 × 10^7^ M^−1^ s^−1^ for CO [64]. Reaction with O_2_ also led to rapid formation of a transient Fe^II^O_2_ complex (λ_max_ 414, 539, 571 nm), followed by autoxidation to the Fe^III^ state within 15 s. On the basis of crystallographic data, the high on-rates exhibited by *Mc*CP-β are attributed to the proximity of the heme cofactor to the protein surface, as well as to the minimal structural rearrangement of distal pocket residues observed upon gas binding. Indeed, the Phe 61 side chain shows no significant conformational changes upon NO or CO coordination, whereas Phe 32 rotates around its Cβ–Cγ bond (Fig. 6) presenting its ring face toward the NO ligand in both subunits of the homodimer, and toward the CO in one of the subunits.

Ligand replacement reactions yielded *k*_off_ values of 0.011 s^−1^ (6c{FeNO}^7^) and 0.20 s^−1^ (Fe^II^CO), which in turn allowed *K*_d_ values of ≤ 1 × 10^–10^ M (6c{FeNO}^7^) and ≤ 8 × 10^–9^ M (Fe^II^CO) to be calculated from the *k*_off_/*k*_on_ ratios (Table 9). The inherently lower heme-gas affinity of the transient Fe^II^O_2_ *Mc*CP-β complex (attributed to the lack of distal pocket hydrogen bond donors) enabled its *K*_d_ value (7.4 × 10^–5^ M) to be determined directly from stopped-flow O_2_ binding data by means of a saturation binding curve. The relatively high off rates for NO and CO are attributed in part to a novel interaction with the Phe 32 aromatic quadrupole. It is proposed that the local negative polarization of the Phe 32 ring face weakens Fe^II^ → XO(π*) backbonding by inhibiting the transfer of electron density to the gas ligand. In support of this hypothesis, RR data point to weaker Fe^II^ → XO(π*) backbonding when the local negative polarity of the Phe 32 aromatic ring face is oriented toward the gas ligand. In the case of the CO complex, doublets of ν(Fe-CO) vibrations (481 and 491 cm^−1^) and ν(CO) vibrations (1971 and 1990 cm^−1^) are observed corresponding to the distinct Fe-CO electrostatic environments evident in subunits A and B of the 6cCO crystal structure. The 481/1990 cm^−1^ frequency combination (typical of a negatively polarized environment) matches subunit A of the Fe^II^CO crystal structure in which the electron rich Phe32 ring face presents toward the CO ligand, whereas the 491/1971 cm^−1^ pair (typical of a neutral environment) matches subunit B in the crystal in which the Phe 32 ring face is oriented away from CO (Fig. 6). The impact of the Phe 32 aromatic quadrupole on heme–CO vibrations is similar to that of nonbonded electrons in the Val68Thr variant of pig Mb [69]. The unusually high ν(NO) frequency (1711 cm^−1^) of the 5c{FeNO}^7^ *Mc*CP-β complex was also attributed to diminished Fe^II^ → XO(π*) backbonding arising from interaction of the NO ligand with the Phe 32 aromatic quadrupole. In this case, only a single ν(NO) mode was observed, suggesting that the Phe 32 ring face presents toward NO in both homodimer subunits, as is the case in the 6c{FeNO}^7^ crystal structure. Evidence that the Phe 32 aromatic quadrupole promotes the release of NO and CO from was obtained by comparing the structural, spectroscopic, and kinetic properties of 6c Fe^II^CO and {FeNO}^7^ complexes of Phe32Val and Phe61Val aromatic → aliphatic variants. X-ray crystal structures and RR spectra of the Phe61Val variant confirm that NO and CO ligands interact with the Phe 32 ring face in a similar manner to that of wt protein. By contrast, the Phe32Val variant shows no influence of any aromatic quadrupole and exhibits a single ν(Fe-CO) RR mode (497 cm^−1^) typical of a neutral heme environment. Consistent with weaker Fe^II^ → XO(π*) backbonding (caused by the Phe 32 aromatic quadrupole), the *k*_off_ values for the CO and NO complexes of wt and Phe61Val *Mc*CP-β are 1.5 (± 0.2)-fold to 3.6 (± 0.3)-fold higher than those of the Phe32Val variant (Table 9). This modest effect is similar to that of nonbonded electrons on CO off rates in the Val68Thr and His64Val/Val68Thr variants of pig Mb that increase the CO off rate by factor of ~ 3 and ~ 4, respectively [69].

## Catalytic activity in cyts P460 and cyts *c* ′-β

### Cyt P460

The catalytic oxidation of NH_2_OH by cyts P460 has been characterized in detail by work from the Lancaster group and others [15, 17, 43, 53]. Within the nitrification pathway of the nitrogen cycle, ammonia is converted to NH_2_OH by either ammonia monooxygenase (AMO) in ammonia-oxidizing bacteria or methane monooxygenase (MMO) in methane-oxidizing bacteria. Cyt P460 is one of two proteins known to carry out the oxidation of NH_2_OH, the second step in the pathway, the other being hydroxylamine oxidoreductase which contains a P460 subunit. The NH_2_OH oxidase activity of purified cyt P460 protein was first reported by Zahn and colleagues with *Mc*P460 having an activity close to that of *Ne*HAO of 366 mol of O_2_/s/mol of enzyme while *Ne*P460 only exhibited an activity of 6 mol of O_2_/s/mol of enzyme [4]. Later activity assays carried out by Lancaster and colleagues under anaerobic conditions using DCPIP as an oxidant helped to demonstrate the importance of key residues within the distal pocket of *Ne*P460 and the inactive *Ns*P460. The wild-type form of *Ne*P460 showed an activity of 4.5 ± 0.1 μM DCPIP·μM^−1^ cyt P460·mM^−1^ NH_2_OH·min^−1^ (Table 10), while the inactive *Ns*P460 had an activity of 0.43 ± 0.02 μM DCPIP·mM^−1^ NH_2_OH·min^−1^ which is consistent with the levels of background consumption seen in the absence of protein (0.44 ± 0.19 μM DCPIP·mM^−1^ NH_2_OH·min^−1^)[43]. Mutation of the *Ns*P460 variant where Ala 131 was replaced with a glutamate residue gave rise to activity around half that of *Ne*P460: 2.1 ± 0.1 μM DCPIP·μM^−1^ cyt P460·mM^−1^ NH_2_OH·min^−1^. Other Ala 131 replacements (Gln, Leu and Asp) resulted in activity levels considered to be equivalent to background consumption (numerical values not reported) [43]. No comparable data for *Mc*P460 have yet been published. Analysis of the products of NH_2_OH oxidation by cyt P460 has also been carried out with Zahn et al. initially demonstrating the production of nitrite under aerobic conditions; however, the stoichiometry of NH_2_OH oxidized to nitrite produced was 1:0.85 suggesting that other products may also have been formed [3]. Caranto and colleagues demonstrated that under aerobic conditions, nitrous oxide was also produced and accounted for the sub-stoichiometry previously reported by Zahn et al. [42]. They also showed that under anaerobic conditions, only nitrous oxide was produced by *Ne*P460 with a stoichiometry of 1 mol of N_2_O per 2 mol of NH_2_OH. This was also corroborated by GC analysis and Griess assays of the Ala131Glu *Ns*P460 mutant which converted NH_2_OH to either NO or N_2_O in the same manner as *Ne*P460 [43]. Again, no such data have yet been reported for *Mc*P460. The Phe41Ala *Ne*P460 mutant demonstrated that production of either N_2_O or NO under aerobic conditions is affected by the residues present in the distal pocket. The fully cross-linked form of the protein demonstrated an activity of  6.4 ± 0.4 μM DCPIP μM^−1^ cyt P460 mM^−1^ NH_2_OH min^−1^ (Table 10) compared to 10.3 ± 0.4 μM DCPIP μM^−1^ cyt P460 mM^−1^ NH_2_OH min^−1^ for the wt protein (a higher activity was attributed to stirring the reaction rather than inverting as was done in the previous measurements). Under anaerobic conditions, the mutant produced nitrous oxide in a manner consistent with the wt protein; however, under aerobic conditions, the amount of nitrite produced dropped to levels much lower than that produced by the wt protein at higher NH_2_OH concentrations with a greater amount of nitrous oxide being produced in its place [59]. The authors concluded that the position of the capping Phe plays an important role in selectivity by providing steric hindrance to the {FeNO}^6^ intermediate during the oxidation of NH_2_OH. Removing the steric hindrance increased the rate of NH_2_OH attack on the {FeNO}^6^ species and ultimately changed the product selectivity toward greater N_2_O production.

**Table 10:** Reported activity in cyts P460

	pH	Activity (µMDCPIP·μM^−1^ cytP460·mM^−1^ NH_2_OH·min^−1^)	Refs.
*Ne*P460 (wt)	8	4.5 ± 0.1	[43]
		10.3 ± 0.4	[59]
(F41A)	8	6.4 ± 0.4	[59]
*Ns*P460 (wt)	8	0.43 ± 0.02	[43]
(A131E)	8	2.1 ± 0.1	[43]
No protein	7	0.44 ± 0.19	[43]

The ability of an enzyme to remove electrons from a heme-bound substrate is highly unusual. Several structural features have been shown to be required for catalysis, notably the cross-link and the presence in an appropriate position of a carboxylate side chain able to participate in proton transfer from heme-bound catalytic intermediates. According to the proposed mechanism, the reaction is initiated by NH_2_OH binding to the high spin ferric heme, which forms a low spin NH_2_OH adduct which is stable in the absence of oxidant. Upon introduction of an oxidant, a new intermediate species is formed with a narrow Soret maximum at 455 nm. A decay product was also witnessed with a less narrow 455 nm Soret maximum and shifted Q-bands. EPR spectroscopy revealed that the decay product signal was consistent with an off path 5c {FeNO}^7^ product, while the intermediate species was EPR silent suggesting that it may be either Fe^II^-NOOH or {FeNO}^6^ [44]. As an Fe^II^-NOOH product would be expected to be competent for anaerobic NO_2_^−^ production, which was not observed, it was concluded that the EPR silent intermediate was {FeNO}^6^. This was supported by the treatment of the resting enzyme with an NO donor to make a “shunted” {FeNO}^6^ product whose spectral features matched that of the oxidant exposed Fe-NH_2_OH species. It is assumed that this species reacts with another NH_2_OH to produce N_2_O and that a final oxidizing equivalent regenerates the ferric enzyme; however, this final step appears to be too rapid to observe.

### *Cyt c′*-β

Remarkably, *Ne*CP-β displays a reactivity to H_2_O_2_ with the formation of a ferryl Fe^IV^ intermediate which is verified by the UV–Vis and EPR spectra [61]. *Ne*CP-β also exhibits a peroxidase-like enzymatic activity with a guaiacol oxidation (*k*_cat_ = 20.0 ± 1.2 s^−1^; *K*_M_ = 2.6 ± 0.4 mM) that is not present in cyt P460 (Table 9). The *k*_cat_ value is considerably lower than that of horseradish peroxidase (441 s^−1^), but the *K*_M_ is of the same order of magnitude between them (3.8 mM for HRP). Interestingly, a mutant of *Ne*P460 Lys70Ala showed a guaiacol dependent peroxidase-like activity unlike wild-type *Ne*P460 [61], indicating that the presence of heme-lysyl cross-link (or the presence of Lys-70) prevents the peroxidase-like activity. This represents an interesting example of how the biological function of the heme protein is altered by the presence or absence of heme-lysyl cross-linking through mutations naturally occurring in AOB.

## Heme-lysine cross-linking mechanism

**Fig. 7:**
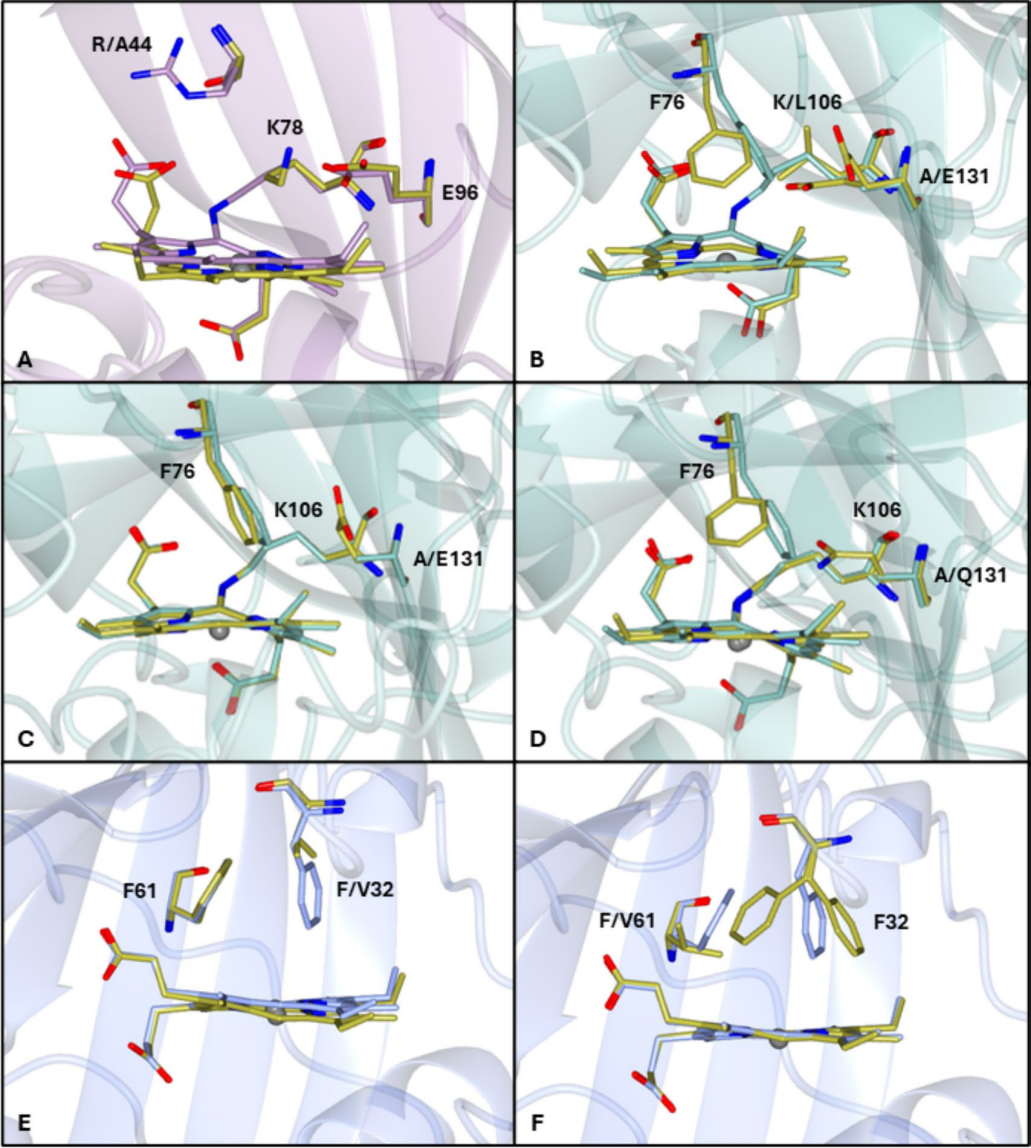
Crystal structures for cyt P460 and *c′*-β mutants (gold) and changes in residue positioning in the distal pocket compared to wt protein (pink – *Ne*P460/green – *Ns*P460/blue—*Mc*CP-β). The *Ne*P460 Arg44Ala mutation (8gar) causes the lysine crosslink to not form in the crystal structure, suggesting that Arg 44 has a role in the formation of the cross-link (**A**). Removal of the cross-link and introduction of a Glu over the distal face of the heme in the *Ns*P460 Lys106Leu/Ala131Glu mutant (6w6n) causes a shift in the positioning of Phe 76 in the distal pocket (**B**). The *Ns*P460 Ala131Glu mutant (6eox) retains its lysine crosslink and causes little movement in Phe 76 with the Glu residue positioned away from the distal heme face (**C**). The *Ns*P460 Ala131Gln mutant (6eoz) retains its lysine crosslink, but the presence of the Gln residue over the distal face of the heme causes Phe 76 to rotate away from the heme face (**D**). Introduction of the *Mc*CP-β Phe32Val mutation (7zs4) causes little change to the other residues in the distal heme pocket (**E**), while the Phe61Val mutation (7zrw) gives rise to two alternative conformations of Phe 32 (**F**)

The formation of the unusual lysine cross-link to the heme of cyt P460 is not well understood, though there is evidence that this can happen autocatalytically as the cross-link spontaneously forms upon aerobic expression of the protein. This may be due to the substitution of the *meso* hydrogen or the mixing of the lone pair on the lysine nitrogen with the π-system of the heme [15]. To investigate the formation of the cross-link, Bollmeyer et al. expressed *Ne*P460 under anaerobic conditions and produced a CLD pro-enzyme [53]. This CLD pro-enzyme was red in color, had spectral features more similar to the cyts *c′*-β (Table 5), and was catalytically inactive. However, treatment of the CLD pro-enzyme with peroxide caused the lysine to form the link and restored the enzyme to its active state. Initial tests of exposing the pro-enzyme to oxygen showed that this alone was not sufficient to form the cross-link and instead resulted in degradation of the protein. In contrast, treatment of the CLD pro-enzyme with Li_2_O_2_, followed by quenching with sodium dithionite and re-oxygenation of the protein with hexaammineruthenium (III) chloride resulted in protein with a spectrum identical to wt *Ne*P460. In the same study, the CLD aerobically expressed Arg44Ala *Ne*P460 mutant was treated with Li_2_O_2_ to investigate if it would form a cross-link. This produced protein which had spectral properties similar to that of cross-linked wt *Ne*P460 protein and regained catalytic activity, although a small proportion of CLD protein remained. A similar effect was seen with a *M*cCP-β Phe61Lys mutant: the anaerobically expressed protein appeared red in color and had spectral features more similar to a CLD P460 than to wt *Mc*CP-β. Treatment with Li_2_O_2_ produced spectral features similar to the Arg44Ala mutant suggesting a mixture of both cross-linked and CLD protein. These data all suggest that a peroxide-dependant post-translation modification is involved in cross-link formation in cyts P460. Both the Arg44Ala and a Phe41Ala *Ne*P460 mutant also provide evidence that other residues in the distal pocket may play a role in cross-link formation. As previously discussed, the Arg44Ala mutant did not form a cross-link when aerobically expressed despite the lysine being present. The structure revealed that the lysine residue had formed a salt bridge with Glu 96 (Fig. 7) suggesting that correctly placed residues within the distal pocket play an important role in ensuring the cross-link is formed. Likewise, the Phe41Ala mutant produced a mixture of cross-linked and CLD protein when expressed anaerobically and catalytic activity was restored upon treatment with Li_2_O_2_ (Tables 9, 10).

Heme distortion occurs even without a lysine cross-link in *Ne*P460, thanks to the interaction between a heme propionate and Arg 44. It can be seen that when this residue is mutated to Ala, the ruffling almost completely disappears (Fig. 7) [53]. This impedes the formation of the lysine cross-link, because it is likely dependent on the oxidation of the *γ-meso* carbon, which is helped by ruffling. Indeed, oxidation can be seen on the opposite *α-meso* carbon of the heme when it is ruffled [50], and in heme oxygenases, oxidation happens on the other two *meso* carbons that are above the plane of the heme because of positive ruffling [53]. However, the equivalent residue in *Mc*P460 (Arg 50) is not placed so as to interact with the heme propionate and yet cross-link formation still occurs. In *Ns*P460, a histidine is in this position which potentially could still interact with the heme propionate. Interestingly, a histidine is also seen in this position in both *Mc*CP-β and *Tt*CP-β but not in *Ne*CP-β. The effect and importance of residues in the distal pocket in relation to the formation of the lysine crosslink and heme ruffling clearly requires further research.

## Summary and outlook

An increasing number of cytochrome P460 and *c′*-β proteins have been characterized in recent years and trends are beginning to emerge that give insight into the relationship between structure and function. The unique cyt P460 cross-link, together with heme distortions and the charged nature of the active site pocket, control the catalytic oxidation of NH_2_OH, whereas the hydrophobic pockets evident in some cyts *c′*-β, along with the lack of a crosslink, are geared toward the reversible binding of nitric oxide. In addition, members of the cyt P460- *c′*-β superfamily that exhibit different types of reactivity remain to be further investigated such as the peroxidase-like enzymatic activity seen in *Ne*CP-β. The tuning of function based on a common overall protein fold may be instructive in the field of protein design where a particular protein scaffold may be modified in pursuit of several different functions or reactivities.

Unanswered questions regarding the mechanisms of cyts P460 and *c′*-β will be addressed in the future by further mutagenesis and ligand-binding studies together with deploying the power of high-performance computing for QM/MM molecular simulations. The reactions of cyt P460 and cyt *c′*-β are well suited to time-resolved investigations using new methods in structural biology. For example, addition of an oxidant to crystals of cyts P460 that have been pre-soaked in NH_2_OH could be used to initiate reactivity that could be followed both structurally and spectroscopically in a time resolved manner.

It remains intriguing why some organisms contain both proteins, while some contain only one. The roles of the proteins may be intertwined to some extent, since the cyt *c′-β* can bind to nitric oxide originating from cyt P460. As more data become available from different organisms, the relationship between environmental factors, nutrient sources, and the presence or absence of certain protein genes may become clearer.

## Data Availability

Crystallographic data for all the structures discussed in this article can be found at the RCSB Protein Data Bank (https://www.rcsb.org/). All other relevant data discussed and reviewed within this article, which include spectroscopic and crystallographic data, are included in this article.

## Abbreviations

AMO: Ammonia monooxygenase
ANB: Ammonia-oxidizing nonlithotrophic bacteria
AOB: Ammonia-oxidizing bacteria
CLD: Cross-link deficient
Cyt: Cytochrome
EPR: Electron paramagnetic resonance
HAO: Hydroxylamine oxidoreductase
*Mc*CP-β: *Methylococcus capsulatus* cytochrome *c*′-β
*Mc*P460: *Methylococcus capsulatus* cytochrome P460
MMO: Methane monooxygenase
*Ne*CP-β: *Nitrosomonas europaea* cytochrome *c*′-β
*Ne*HAO: *Nitrosomonas europaea* hydroxylamine oxidoreductase
*Ne*P460: *Nitrosomonas europaea* cytochrome P460
NiR: Nitrite reductase
NOR: Nitric oxide reductase
*Np*CP-β: *Nitrosospira sp.* *NpAv* cytochrome *c*′-β
*Ns*P460: *Nitrosomonas* *sp.* AL212 cytochrome P460
NSD: Normal coordinate structural decomposition
*Tt*CP-β: *Thermus thermophilus* cytochrome *c*′-β
5c: Five-coordinate
6c: Six-coordinate
